# LRR-RLK subfamily II of coreceptors: emerging, non-canonical and canonical roles in plant antiviral immunity and development

**DOI:** 10.3389/fpls.2025.1694090

**Published:** 2025-11-17

**Authors:** Fellipe R. Sampaio, Beatriz M. Takagaki, Sâmera S. Breves, Raquel G. Rodrigues, Viviano G. O. Neves, Félkerson M. Ferreira, Nathália G. A. Ribeiro, Eulálio G. D. Santos, Pedro A. B. Reis, Elizabeth P. B. Fontes

**Affiliations:** Department of Biochemistry and Molecular Biology, BIOAGRO, National Institute of Science and Technology in Plant-Pest Interactions, Universidade Federal de Viçosa, Viçosa, MG, Brazil

**Keywords:** NSP-interacting kinase, SERK, CIK, coreceptors, receptor-like kinases, antiviral immunity, signaling hubs

## Abstract

Coreceptors act together with receptors in the process of signal transduction. Within the LRR-RLK subfamily II, coreceptors play an essential role by serving as a connection between growth and immunity in plants. The 14 LRRII-RLK identified genes in Arabidopsis have been phylogenetically clustered in four closely related groups. Three of them have been functionally characterized: (i) NIKs, which are associated with responses to viral infections, (ii) SERKs, which are involved in both development and immunity, and (iii) CIKs, which are connected to homeostasis, growth, and meristem development, as well as to a lesser extent, immunity. Currently, LRRII-RLKs have been more intensively investigated as potential antiviral mechanisms due to their emerging roles in antiviral immunity and their potential of being targeted by viral manipulation. Despite their partial functional redundancy and interactions in immunity and developmental signaling mechanisms, targeting LRRII-RLKs through genetic manipulation may lead to the development of a broad-spectrum resistance to viral infections, while also preserving plant growth and yield.

## 1 Introduction

The RLK (Receptor-Like Kinase) superfamily is the largest group of receptor proteins found in plant cells. In *Arabidopsis thaliana*, there are over 600 RLKs in this superfamily, making up around 2.5% of all protein-coding genes (Altschul et al., 1997; Escocard de Azevedo Manhães et al., 2021). These receptors are essential to the molecular mechanisms that enable plants to perceive, transmit, and integrate a diverse range of environmental and endogenous signals into physiological responses. Therefore, their diversity may be an evolutionary adaptation to the sessile lifestyle of plants, enabling them to modulate development, cell differentiation, immunity, and responses to biotic and abiotic stress.

**Figure 1:**
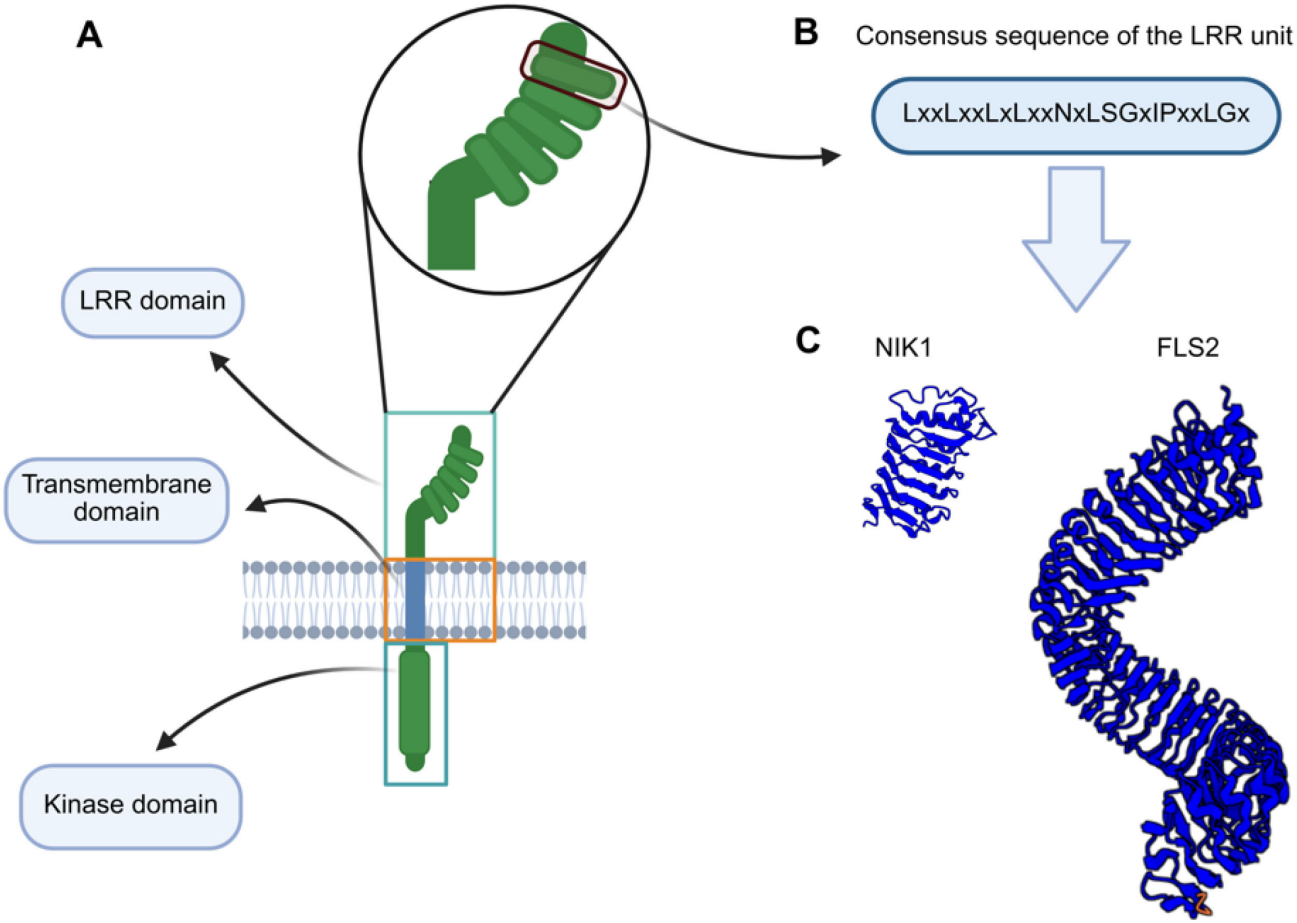
Schematic representation of the LRR-RLK domains. **(A)** Members of the LRR-RLK family have a tripartite receptor configuration. They share (i) a more divergent LRR ectodomain, responsible for specific ligand recognition and dimerization with the cognate coreceptor, (ii) a single-pass transmembrane segment that anchors the protein into the membrane, and (iii) a conserved cytoplasmic serine-threonine domain that transduces the external signal to the intracellular environment. **(B)** Consensus sequence of an LRR motif (unit). **(C)** LRR ectodomains of representative members of the LRR-RLK family. The NIK1 ectodomain harbors 5 LRR motifs, while the FLS2 ectodomain contains 29 LRR motifs.

Canonical RLKs exhibit a typical tripartite structure that consists of a divergent ligand-binding extracellular domain (ectodomain), a single-pass transmembrane helix, and a conserved cytoplasmic serine/threonine kinase domain, which transduces signals through phosphorylation cascades (**Figure 1**) (Shiu and Bleecker, 2001; Escocard de Azevedo Manhães et al., 2021). RLKs recognize specific ligands that drive their dimerization with coreceptors (CoRK), thereby activating the RK-CoRK complex by phosphorylating one another. The activated RK-CoRK complexes relay signals through interactions with adaptor proteins and cytoplasmic modulators, resulting in the regulation of development, cell differentiation, and defense mechanisms against pathogens and damage caused by biotic and abiotic factors (Shiu and Bleecker, 2001; Ferreira et al., 2025; Heinlein, 2025).

The leucine-rich repeat (LRR) ectodomain-containing RLKs constitute the major family of the RLK superfamily, containing 38% of the identified Arabidopsis RLK members. They are essential regulators of plant immunity and development (Jamieson et al., 2018). This LRR-RLK family is further divided into at least 13 major subfamilies, with additional subdivisions in some groups (e.g., VI, VII, XIII), resulting in 19 functional clades (Liu et al., 2017). Structural diversity in numbers and arrangement of LRR motifs (3 to 26) within the ectodomain contributes to ligand specificity across subfamilies (Shiu and Bleecker, 2001; Shiu et al., 2004; Chen, 2021) (**Figure 1**). LRR-RLKs are functionally classified as primary receptors or coreceptors. The receptors are characterized by their typical long LRR domains and specialized ligand-binding ectodomains. In contrast, the coreceptors contain fewer LRRs and participate in stabilizing receptor-ligand interactions and signal amplification, while maintaining the specificity of the cognate receptor-induced response (Xi et al., 2019).

**Figure 2:**
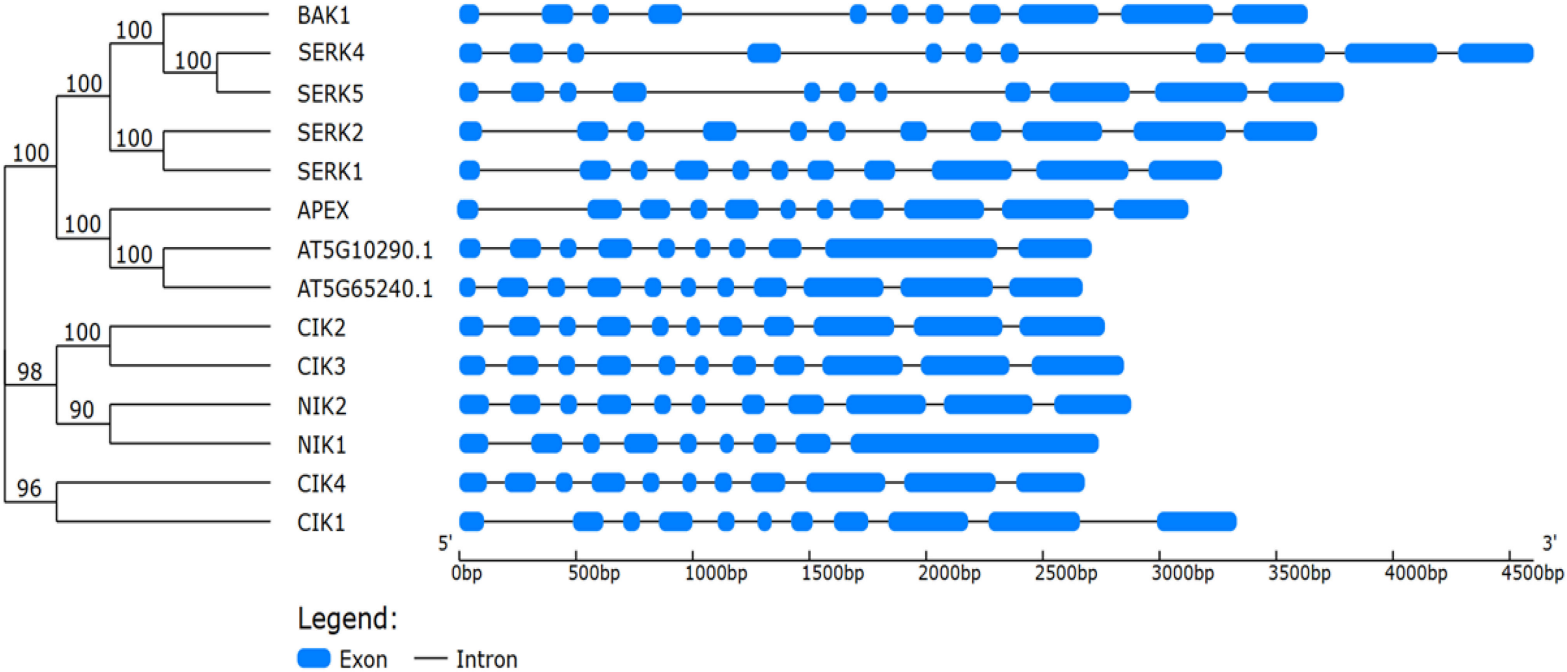
Phylogenetic relationships and gene structure organization of *Arabidopsis thaliana* LRR-RLK subfamily II members. The phylogenetic tree (left) was constructed using the protein sequences of the 14 LRR-RLK subfamily II members from *A. thaliana*, with bootstrap support values (%) shown at the nodes. The corresponding exon–intron structures (right) were determined based on genomic and coding DNA sequences retrieved from the TAIR database. The exons are represented by blue boxes and introns, by black lines, scaled according to nucleotide length (bp). The analysis highlights the conservation and variation in exon–intron organization among different clades, which include SERKs, CIKs, and NIKs.

The LRR-RLK subfamily II (LRRII-RLK) is represented by a structurally conserved yet functionally diverse monophyletic group that is prevalent in several species within the plant kingdom, including economically relevant crops (**Supplementary Figure 1**; https://itol.embl.de/export/20023519899154591760355171) (Sakamoto et al., 2012; Hosseini et al., 2020). Comparative phylogenies suggest that LRRII-RLKs were already present in ancestral angiosperms and later underwent expansions driven by gene duplication and selective pressures (Shiu et al., 2004; Sakamoto et al., 2012). This interpretation is supported by the similar organization of introns/exons of closely related genes (**Figure 2**). Members of subfamily II of RLKs have been grouped based on sequence conservation and shared structural properties, forming well-supported clades, such as Group NIK [NUCLEAR SHUTTLE PROTEIN (NSP)-INTERACTING KINASE], containing NIK1 and NIK2 sub-clades, Group CIK (CLAVATA3 INSENSITIVE RECEPTOR KINASE), comprising CIK2/3, CIK1 or NIK3, and CIK4 subclades, Group LRR2c and Group SERK (SOMATIC EMBRYOGENESIS RECEPTOR-LIKE KINASE), subdivided in the subclades SERK1/2, SERK3 or BAK1 [BRASSINOSTEROID INSENTIVE-1 (BRI1)-ASSOCIATED RECEPTOR KINASE], SER4/5 (SERK5, a pseudogene) (**Figure 2**; **Supplementary Figure 1**).

In *Arabidopsis thaliana* and *Solanum lycopersicum*, the NIK clade includes receptor kinases first implicated in antiviral defenses (Calil and Fontes, 2017; Teixeira et al., 2019), while the SERK clade contains receptor kinases involved primarily in plant immunity, brassinosteroid (BR) signaling, and somatic embryogenesis (Liu et al., 2020). Members of the CIK clade are involved in development, and some evidence suggests a role in plant immunity (Wu et al., 2020; Zhu et al., 2021). In contrast, the LRR2c clade comprises proteins whose biological functions have yet to be fully characterized (Sakamoto et al., 2012; Liu et al., 2017). The 14 members of the LRRII-RLK subfamily in Arabidopsis are partially described in the Arabidopsis Information Resource (TAIR) database and modified in **Supplementary Table 1**. The LRRII-RLK members share a compact extracellular domain with five LRR motifs and a well-conserved intracellular kinase domain. The LRR-RLKII proteins act as coreceptors, forming heterodimers with ligand-binding RLKs or RLPs (Receptor-Like Proteins) to regulate stimulus-specific signaling pathways and/or as signaling hubs to control stress-shared transduction pathways (Fontes, 2024).

As shared properties, LRRII-RLK coreceptors and signaling hubs serve as substrates for ligand-specific receptors and are activated by cognate receptor-induced phosphorylation. However, they diverge in some aspects. Upon ligand binding to the receptor, the coreceptor interacts physically with its cognate receptor, resulting in phosphorylation-induced activation and subsequent signaling. Furthermore, the coreceptor stabilizes ligand binding to the receptor and is essential for receptor function and signaling (Xi et al., 2019). Remarkably, the coreceptors are flexible in receptor interaction, while maintaining the receptor specificity of the signaling pathway. In contrast, the signaling hub may not interact directly with a stimulus-specific receptor and acts as a downstream component to which converge the transduction of different signals into a unified physiological response (Fontes, 2024). Typical examples of coreceptors are SERKs (Liu et al., 2020), and, more recently, NIKs have emerged as signaling hubs (Fontes, 2024; Ferreira et al., 2025).

Here, we describe the functional roles of members of the LRRII subfamily and highlight newly discovered immunological strategies employed by plants against viruses. We also describe the intricate crosstalk of these antiviral responses with pattern-triggered immunity (PTI). Furthermore, we discuss the role of LRRII-RLK as a possible link in the relationship between growth/development and plant antiviral immunity.

## 2 The NIK cluster: NIK1 and NIK2

The constant exposure of plants to various biotic and abiotic stressors has driven the evolution of sophisticated signaling networks that regulate development and immune responses (Hu et al., 2018; Teixeira et al., 2019; Zhou and Zhang, 2020). The binding of pathogen-associated molecular patterns (PAMPs) to pattern recognition receptors (PRRs) induces PRR interaction with members of the LRRI-RLK subfamily, initiating signal transduction. This ligand-induced formation of the activated PRR-LRRIIRLK complex is the foundation of PTI (Matsubayashi, 2003). Like other RLRII-RLKs, NIKs have emerged as critical mediators of signal perception and integration at the plasma membrane (Mariano et al., 2004; Ferreira et al., 2025). However, NIK1 and NIK2 were first characterized by their redundant roles in antiviral signaling, a non-canonical type of PTI (Li et al., 2019a).

### 2.1 NIK1 and NIK2 in antiviral immunity

The NIK1 protein was initially identified by its interaction with NSP from tomato-infecting begomoviruses (Fontes et al., 2004; Mariano et al., 2004). Subsequent studies identified three Arabidopsis closely related genes, designated NIK1 (AT5G16000), NIK2 (AT3G25560), and NIK3 (AT1G60800), which also interacted with NSP from cabbage leaf curl virus (CabLCV), an Arabidopsis-infecting begomovirus. NIK1 is a functional kinase-type protein with autophosphorylation capability, a feature that facilitates its self-regulation and role as a potential membrane coreceptor and/or a signaling hub (Santos et al., 2010; Fontes, 2024). Several lines of evidence suggest that NIK1 is involved in plant defense. Firstly, NIK1 is a virulence target of the viral suppressor NSP (Fontes et al., 2004). Additionally, the loss of *NIK1* and *NIK3* function is associated with an increased susceptibility phenotype to begomovirus infection (Fontes, 2024; Rocha et al., 2008; Santos et al., 2009). Furthermore, the constitutive activation of the NIK1 antiviral signaling by expressing a phosphomimetic NIK1, NIK1-T474D, in Arabidopsis and tomato plants enhances resistance to begomovirus (Brustolini et al., 2015; Zorzatto et al., 2015). Further characterization of the NIK1 signaling includes the identification of viral PAMPs, RNA and DNA extracted from infected plants, that activate NIK1, as well as the RIBOSOMAL PROTEIN RPL10 and L10-INTERACTING MYB DOMAIN-CONTAINING PROTEIN (LIMYB) downstream components, which are phosphorylated upon NIK1 activation (Carvalho et al., 2008; Zorzatto et al., 2015; Teixeira et al., 2019; Li et al., 2019a).

**Figure 3:**
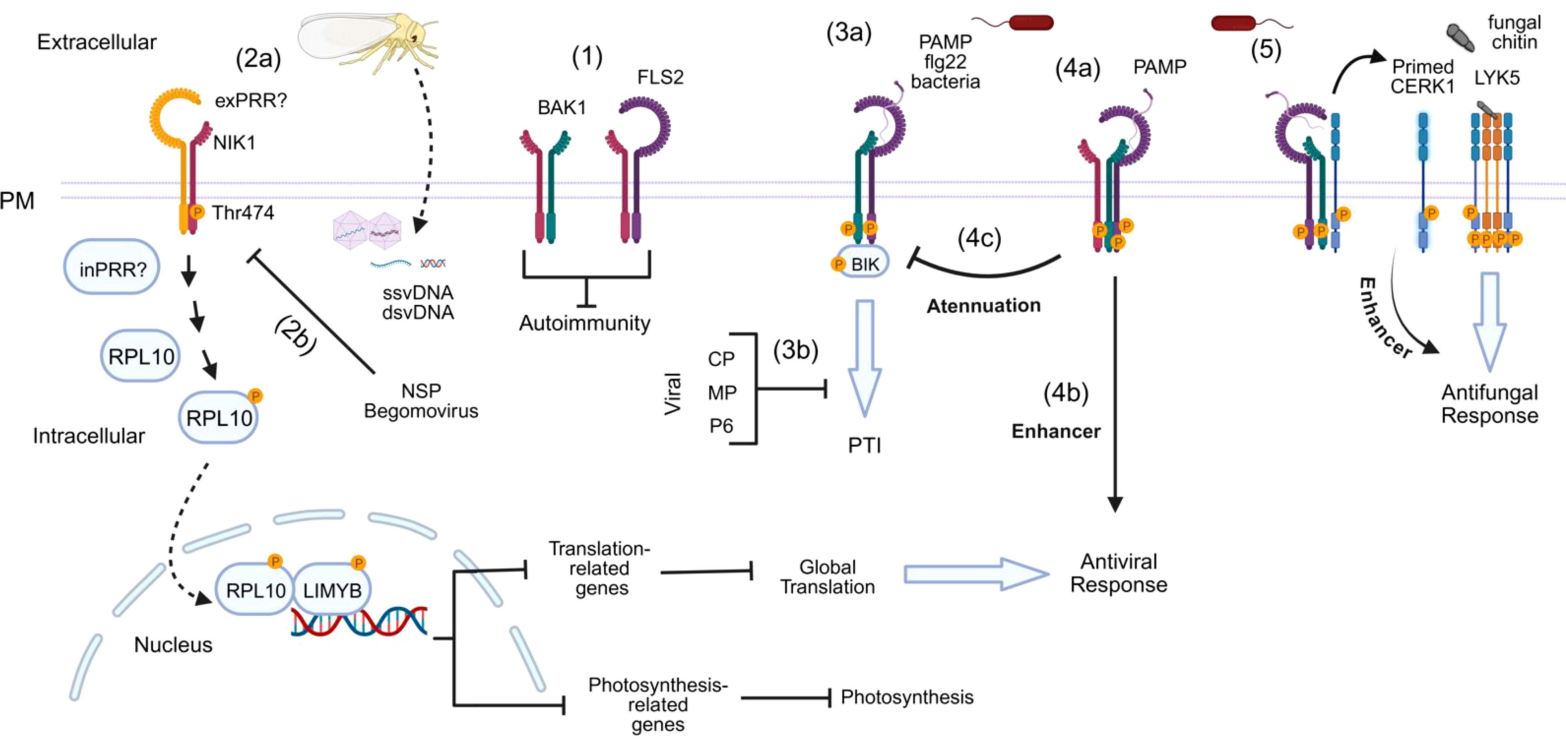
Interplay between coreceptors in response to different biotic stimuli. **(1)** NIK1 associates with FLS2 and/or BAK1 to prevent autoimmunity in the absence of pathogen invasion. **(2a)** Upon white-fly transmitted-begomovirus infection, a possible transmembrane (exPRR) or intracellular (inPRR) Pattern Recognition Receptor (PRR) detects the viral signal and activates the NIK1-RPL10-LIMYB antiviral pathway. This pathway involves a phosphorylation cascade initiated by NIK1 at threonine 474, leading to RPL10 phosphorylation and, subsequently, LIMYB phosphorylation in the nucleus. LIMYB acts as a transcription factor that represses genes involved in the global translation machinery and photosystem genes. Repression of translation-related genes leads to inhibition of global translation and, consequently, viral infection. **(2b)** The NSP of begomoviruses binds to the kinase domain of NIK1 to prevent phosphorylation, suppressing the antiviral mechanism. **(3a)** Canonical PTI. After bacterial infection, flg22 is recognized by the immune receptor FLS2, which recruits BAK1 and BIK1. Phosphorylation events initiate immune signaling, and BIK1 is released from the complex to activate downstream signaling. **(3b)** Viral proteins such as Coat Protein (CP), Movement Protein (MP), and Protein P6 can inhibit PTI responses. **(4a)** NIK1 remains bound to the flg22-induced FLS2-BAK1 complex and, then, activated BAK1 phosphorylates NIK1 and **(4b)** thus activates the RPL10-LIMYB antiviral pathway. **(4c)** Phosphorylated NIK1 attenuates PTI or antibacterial response. **(5)** The activation of BAK1 also triggers the phosphorylation of CERK1 in its intracellular juxtamembrane region, priming the host for potential fungal attacks. This process is mediated by the CERK1-LYK5 complex that recognizes fungal PAMP (chitin).

The current mechanistic model of the NIK1 antiviral signaling pathway suggests that in the presence of begomovirus-derived nucleic acids, which function as viral PAMPs, NIK1 heterodimerizes with an unknown viral pattern recognition receptor (vPRR), causing sequential phosphorylation at threonine residue 474 to activate the pathway and at threonine 469 as a form of autologous regulation (**Figure 3**) (Santos et al., 2010; Teixeira et al., 2019). As any other virus, begomoviruses are obligate intracellular parasites; thereby, a putative extracellular vPRR may undergo endocytosis to recognize vPAMP intracellularly. Alternatively, vPAMP may be recognized by an intracellular vPRR. Upon activation, NIK1 mediates the phosphorylation of RPL10 at Ser, position 104 (Carvalho et al., 2008; Rocha et al., 2008), which is, in turn, relocated to the nucleus where it interacts with the LIMYB transcription factor (Zorzatto et al., 2015). LIMYB has also been shown to be activated by NIK1-mediated phosphorylation at positions 157, 161, and 162 (Ferreira et al., 2025). The activated RPL0-LIMYB complex fully represses the expression of ribosomal protein genes (RP) and translation-related genes, resulting in the suppression of global mRNA translation (Zorzatto et al., 2015). More recently, the activation of the NIK1/RPL10/LIMYB signaling module has also been demonstrated to repress the expression of photosynthesis-related genes and photosynthesis itself (Ferreira et al., 2025). Therefore, NIK1 signaling coordinates the regulation of translation and photosynthesis.

In begomovirus-infected cells, the viral genome is unpacked in the cytoplasm. The capsid protein (CP)-bound single-stranded viral DNA (ssvDNA) interacts with an importin and is translocated to the nucleus. In the nucleus, ssvDNA is converted into a double-stranded intermediary (dsvDNA) to replicate the viral genome and transcribe viral genes (Teixeira et al., 2019, 2021; Breves et al., 2023). The NIK1-mediated pathway activation by viral PAMPs results in a decreased association of viral mRNA with the polysomes and hence impairs the viral mRNA translation (Brustolini et al., 2015; Zorzatto et al., 2015). However, this plant antiviral mechanism has been evolutionarily overcome by begomoviruses because NSP binds to the activation loop of NIK1, preventing the phosphorylation of Thr-474 and subsequent activation of the defense pathway (Fontes et al., 2004; Santos et al., 2009). Thus, by allowing the translation to proceed, NSP effectively evades plant immunity. Furthermore, by blocking this pathway, the virus also avoids the repression of genes associated with photosynthesis and growth (Ferreira et al., 2025).

Despite the downstream events of NIK1 antiviral signaling being dissimilar to those of canonical PTI, which often involve reactive oxygen species (ROS) accumulation, MAPK phosphorylation and activation, upregulation of typical defense genes against pathogens, and callose deposition (Bigeard et al., 2015), the upstream events of NIK1 signaling and PTI are similar (Santos et al., 2009). For instance, NIK1 is structurally related to the SERK PTI coreceptors, and they share a highly conserved kinase domain and activation loop. Furthermore, NIK1 activation requires phosphorylation at the crucial Thr-474 residue, which conserves a similar position to the activation site within the activation loop of SERKs; thereby, suggesting a similar activation mechanism. Additionally, like PTI, NIK1 is induced by PAMPs (**Figure 3**) (Teixeira et al., 2019). Finally, the NIK1 antiviral signaling is suppressed by the begomoviral NSP, similarly to PTI, which can also be inhibited by other viral suppressor proteins, including CP from plum pox virus (PPV), MP from cucumber mosaic virus (CMV), and P6 from cauliflower mosaic virus (CaMV) (Kørner et al., 2013; Zvereva et al., 2016; Nicaise and Candresse, 2017). Therefore, based on similar PTI upstream mechanisms for signaling activation and suppression, NIK1 antiviral signaling may be considered as a non-canonical type of PTI.

The functional redundancy of NIK1 and NIK2 in antiviral mechanisms has been monitored through reverse genetics studies. Although the loss of NIK2 function has been previously demonstrated not to affect begomoviral infection (Fontes et al., 2004), further studies have challenged this result. Like *NIK1*, *NIK2* gene expression is upregulated by begomoviral infection (Zorzatto et al., 2015). Additionally, viral PAMP-induced repression of translation- and photosynthesis-related genes is attenuated in *nik1–1* and *nik2–1* knockout lines and totally abolished in *nik1-1nik2–1* double mutant (Ferreira et al., 2025). Furthermore, RNA and DNA extracted from CabLCV-infected plants mediate a rapid phosphorylation of NIK1 in wild-type and *nik2–1* lines but not in the double mutants (*nik1-1nik2-1)* (Ferreira et al., 2025). Finally, an interaction network at the cell surface based on LRR identified NIK2 as a key node in the information spread, showing strong connectivity with NIK1 | (Li et al., 2019a). This finding further supports the hypothesis of functional redundancy, despite NIK1 playing a prominent role in signal dissemination within the network (Li et al., 2019a). Collectively, these results confirm that *NIK1* and *NIK2* are paralogs.

### 2.2 NIK1 and NIK2 inhibits PTI, yet NIK1 is phosphorylated by the FLS2-BAK1 immune complex to activate antiviral signaling

NIK1 and NIK2 are involved in plant defense against begomovirus (Mariano et al., 2004; Carvalho et al., 2008; Zorzatto et al., 2015; Teixeira et al., 2019; Li et al., 2019a; Ferreira et al., 2025) while simultaneously acting as a negative regulator of bacterial PTI (Li et al., 2019a). The first evidence demonstrating that NIK1 inhibits antibacterial PTI was derived from genetics studies in which the inactivation of *NIK1* enhanced bacterial resistance and increased PTI response in the knockout lines, a phenotype restored to wild-type levels by *NIK1* complementation (Ahmed et al., 2018; Li et al., 2019a). The *nik1–1* mutant displays resistance to *Pseudomonas syringae* (Pst DC3000 and Psm ES4326), with reduced bacterial growth and milder disease symptoms. Although *nik2–1* displays a moderate increase in resistance to bacteria, the double mutant *nik1-1/nik2–*1 was no more resistant than *nik1–1* alone. In contrast, APEX (AT5G63710, an LRR2c member) knockout lines exhibit increased susceptibility to *P. syringae pv. tomato* DC3000 (Pto DC3000), consistent with a positive role in antibacterial defense (Ahmed et al., 2018; Smakowska-Luzan et al., 2018). These results suggests that NIK1 and NIK2 act as negative regulators of PTI, while APEX acts antagonistically, highlighting the functional diversity within the LRRII-RLK subfamily in modulating plant immunity.

Additional quantitative protein-protein assays indicated that NIK1 negatively regulates the formation of the bacterial PAMP (flg22)-induced PTI complex, formed by the FLAGELLIN-SENSITIVE 2 (FLS2) PRR and its cognate coreceptor BAK1 (Li et al., 2019a). Furthermore, flg22 perception by FLS2 induces NIK1 and RPL10 phosphorylation, which are readouts of the NIK1-antiviral signaling pathway activation. Phosphorylated NIK1 remains bound to the FLS2-BAK1 complex, attenuating the PTI response. *In vitro* phosphorylation assays coupled to MS analysis demonstrated that BAK1 phosphorylates NIK1 at the activation phosphosite, Thr-474. Collectively, these data, along with functional genomics, have uncovered the underlying mechanism for the NIK1 interplay between canonical PTI and antiviral signaling (Li et al., 2019a). Under normal conditions, NIK1 is associated with the FLS2 receptor and the BAK1 coreceptor to prevent autoimmunity (**Figure 3**). During bacterial infection, the bacterial PAMP flg22 is recognized by FLS2, which recruits BAK1 to form an activated immune complex to initiate PTI. NIK1 remains bound to the immune complex and so is phosphorylated by activated BAK1 at Thr-474, attenuating PTI and relaying an antiviral signal to the RPL10/LIMYB signaling module to suppress global translation and reduce photosynthesis (**Figure 3**). Therefore, FLS2:BAK1-induced phosphorylation of NIK1 does not result in a host priming state for subsequent viral infection but rather in activation of an antiviral mechanism that protects plants against begomoviruses, one of the largest groups of plant virus (Fiallo-Olivé and Navas-Castillo, 2023). The NIK1-inverse modulation of antiviral and antibacterial immunity enables bacteria and viruses to activate immune responses against each other, probably to prevent multiple infections and thus competition.

### 2.3 NIK1/NIK2: integrative immune hubs modulating translation, photosynthesis, stress responses, and growth

**Figure 4:**
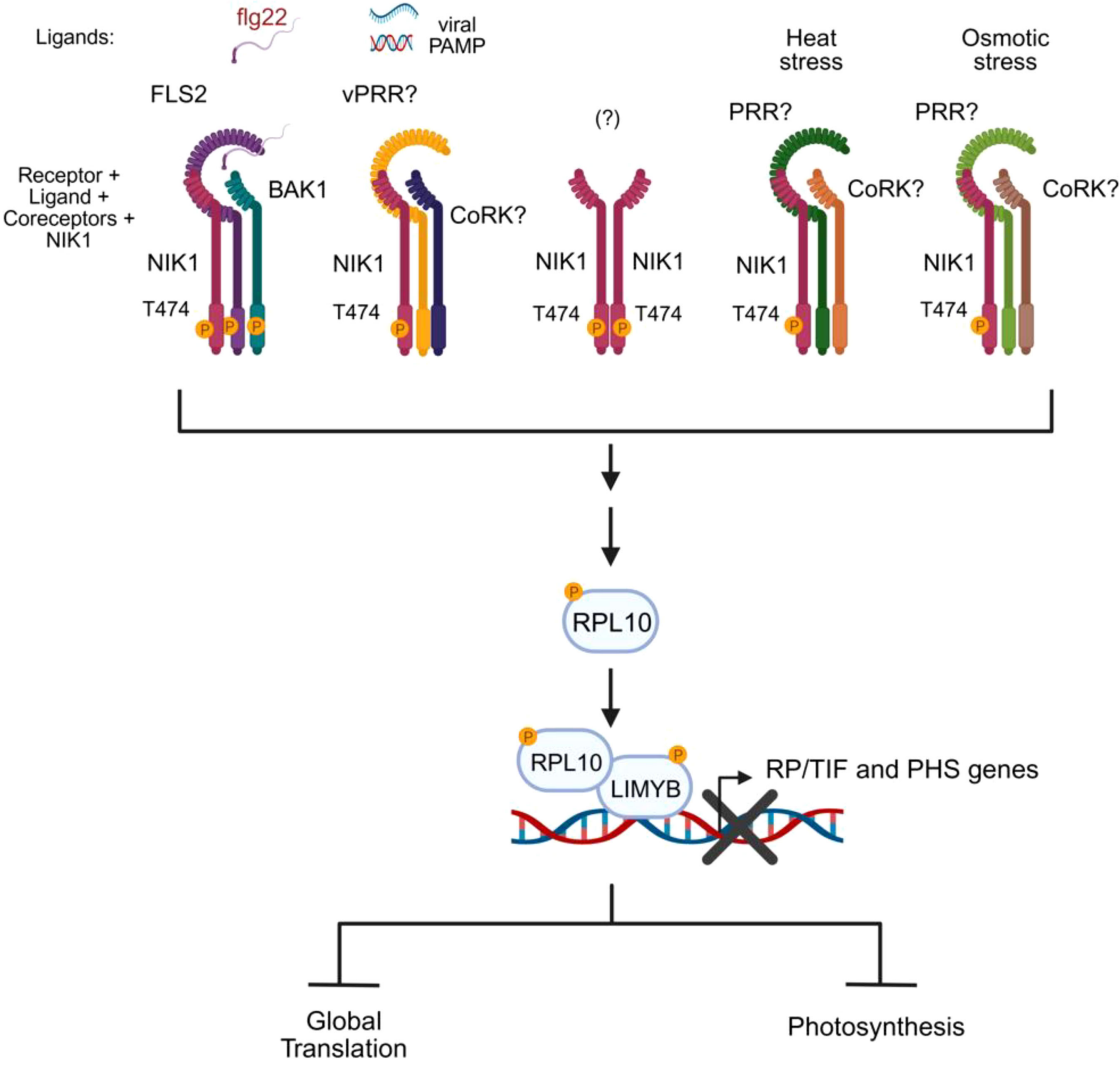
NIK1 serves as a signaling hub activated by biotic and abiotic signals. NIK1 is phosphorylated and activated by specific stress-sensing receptors that, upon perception of biotic or abiotic stress signals, form active complexes with their cognate coreceptors. Activated NIK1 relays the converged stress signals to a unified signaling circuit (RPL10/LIMYB), leading to a shared physiological response, translation suppression, and photosynthesis reduction. The question marks denote the unknown components of the signaling module. The essential phosphorylation site for activation is indicated in the kinase domain. vPRR, viral PAMP recognition receptor; CoRK, coreceptor kinase; RP, ribosomal protein; TIF, translation initiation factor; PHS, photosystem-related genes.

The transcriptional landscape of LIMYB, along with genetics and biochemical studies, confirmed that the NIK1/NIK2-RPL10-LIMYB signaling module coordinates the regulation of translation and photosynthesis under biotic and abiotic stress conditions (Ferreira et al., 2025). In addition to viral and bacterial PAMPs, both heat and osmotic signals induce NIK1 phosphorylation and activate the NIK1 signaling in a LIMYB-dependent manner (**Figure 4**). Therefore, LIMYB links NIK1 activation to the repression of translation- and photosynthesis-related genes, leading to the suppression of global translation and the reduction of photosynthesis, presumably to balance carbon supply and demand under stress conditions (Ferreira et al., 2025). LIMYB-overexpressing lines and Arabidopsis ectopically expressing the phosphomimetic NIK1-T474D mutant line display stunted vegetative growth, smaller roots, and reduced germination, which is consistent with decreased translation and photosynthesis (Zorzatto et al., 2015; Ferreira et al., 2025). Nevertheless, the constitutive activation of NIK1 by expressing NIK1-T474D improves drought tolerance in Arabidopsis lines (Ferreira et al., 2025). Mechanisms, which balance energy production with resource allocation under stress conditions, such as NIK1-mediated coordinate regulation of translation and photosynthesis, may also aid in mitigating oxidative stress buildup and confer a certain level of stress tolerance.

The transduction of stress signals by the NIK1-RPL10-LIMYB signaling module holds similar, but also different characteristic from other stress-induced signaling pathways (Fontes, 2024). Similar to other signaling pathways, the sensing receptors are expected to be very specific for their cognate stimulus. While the bacterial PAMP flg22 induces NIK1 signaling activation via the sensing receptor FLS2 and its coreceptor BAK1, the perception of viral PAMPs (begomovirus-derived nucleic acids) does not require the FLS2-BAK1 immune complex to activate the NIK1-mediated antiviral signaling and hence may require a yet-to-be-determined viral PAMP recognition receptor (Li et al., 2019a; Ferreira et al., 2025) (**Figure 4**). Likewise, signaling receptors recognize stimuli with high specificity and affinity, which precludes a single receptor from being activated by multiple signals. Therefore, abiotic stresses (heat and osmotic stress) employ different stress-sensing receptors to activate the NIK1-RPL10-LIMYB module. However, differently from other signaling pathways, which often transduce specific stimuli into specific responses, the NIK1 signaling serves the same signaling circuit (RPL10-LIMYB) to relay different stress signals into a unified physiological response (**Figure 4**). Therefore, NIK1 acts as a signaling hub to which distinct stimuli converge to a shared response.

## 3 The SERK clade

SERKs are involved in a wide range of processes, including immunity, control of cell death, BR-mediated growth, floral organ abscission, root meristem growth, and stomatal patterning (Ma et al., 2016; Liu et al., 2020). Except for the *SERK5* pseudogene, the other four SERKs of subfamily II of LRR-RLKs, *SERK1*, *SERK2*, *SERK3*, and *SERK4*, have been extensively reviewed (Ma et al., 2016; He et al., 2018; Kumar and Van Staden, 2019).

### 3.1 From development to cell death: the redundancies and specializations of functions

SERKs, the LRRII-RLKs essential for somatic embryogenesis, were first identified in Daucus carrot cell cultures during the embryogenesis phase (Schmidt et al., 1997). Their recent application in the rice callus induction further confirmed their role in embryogenesis (Mostafiz and Wagiran, 2024).

*SERK1/2* are expressed primarily in flowers and seeds, while *SERK3* is abundantly expressed in most tissues, and *SERK4* is most highly expressed in mature leaves (Colcombet et al., 2005; Li et al., 2019b). All SERKs are also present in meristems. SERK3 (BAK1) and SERK1 function as coreceptors for the BRI1 receptor, responsible for BR perception and signaling. Previous studies have demonstrated that SERK3 and its homologs, including SERK1, SERK2, and SERK4, are functionally redundant in the BRI1 receptor signaling pathway (Chinchilla et al., 2007; He et al., 2007). However, only SERK3/BAK1 is essential for activating the BRI1 receptor and the subsequent downstream events in BR signaling (Gou et al., 2012).

SERK1/2 also exhibit functional redundancy in regulating cell differentiation, the vascular system organization, and the male gametophyte development during the early stages of anther formation, acting as coreceptors of EMS1 (EXCESS MICROSPOROCYTES1) (Li et al., 2017). The peptide TPD1 (TAPETUM DETERMINANT1) binds to the heterodimer formed by EMS1/SERK1/2, leading to complex activation through kinase phosphorylation. Subsequently, the BRI-1-EMS-SUPRESSOR1 (BES1) transcription factor is activated to control tapetum development, which is a nutritive layer surrounding the anther that nourishes the developing pollen. This process is essential for pollen biogenesis (Chinchilla et al., 2007; Li et al., 2017; Chen et al., 2019; Yan et al., 2025).

*SERK1/2/3/4* genes, with their overlapping functions, are also involved in regulating the floral abscission process. They act as coreceptors of HAESA and HAESA-LIKE 2 (HSL2) sensory receptors, which recognize the IDA (INFLORESCENCE DEFICIENT IN ABSCISSION) peptide (Meng et al., 2016). This process is crucial for removing external floral parts, such as sepals, petals, and stamens, after fertilization of the internal gynoecium.

*SERK1/2/3* genes also participate in root meristem growth, as demonstrated by the very short roots and reduced meristematic cortex observed in the triple mutant (Ou et al., 2022). The ROOT MERISTEM GROWTH FACTOR1 (RGF1) is perceived by a set of functionally redundant RGF1 INSENSITIVEs (RGIs)/RGF RECEPTORs (RGFRs) that undergoes ligand-induced hetero dimerization with their cognate coreceptor BAK1 and/or SEK1/2. The RGF1-RGIs-SERK complex regulates the expression of PLETHORA 1 (PLT1) and PLT2 for controlling root meristem activity via YODA, MKK4/MKK5, and MPK3/MPK6 signaling cascades (Lu et al., 2020; Shao et al., 2020). This phenomenon is independent of the BRI1 pathway because, although MAPK3 and MAPK4 phosphorylation levels increase in the presence of ROOT MERISTEM GROWTH FACTOR1 (RGF1), the YODA-MKK4/5-MPK3/6 module is activated in *bri1* mutants, but not in *serk* or *rgf1* mutants (Ou et al., 2022).

SERK4, also known as BKK1 (BAK1-like kinase 1), and SERK5 share a high degree of conservation with SERK3 (BAK1). However, in contrast to *SERK4* and *SERK3*, *SERK5* is unable to restore the *bri1–5* mutant phenotype (He et al., 2007). The Col-0 version of SERK5 has an amino acid substitution at position 401, located within the kinase RD motif (arginine/aspartate) (He et al., 2007), The replacement of arginine (Arg) with leucine (Leu) blocks the catalytic activity of the kinase, resulting in a non-functional form of SERK5 in the BR signaling pathway.

Together with SERK3, SERK4 participates in controlling cell death, but independently from the BR signaling pathway (He et al., 2007; Li et al., 2019b). BAK1 and BKK1 have a positive effect on BR-mediated cell growth, while negatively regulating a cell death pathway that is associated with immunity. *bak1–4 and bkk1–1* mutant lines exhibit a constitutively activated cell death pathway, which causes spontaneous cell death in plants (He et al., 2007). This cell death phenotype suggests that these proteins play a role in maintaining cellular homeostasis by modulating responses to the presence or absence of specific signals.

The functional catalogue of SERKs demonstrates a finely tuned balance between redundancy and specialization. This balance enables the SERK coreceptors to participate in a broad spectrum of developmental programs while contributing specifically to distinct signaling pathways (Ma et al., 2016; Hohmann et al., 2017; Gou et al., 2012). This plasticity is highlighted by the overlapping roles in BR signaling, floral abscission, and meristem maintenance, along with more specialized functions, including the unique catalytic limitations of SERK5 or the SERK3- or SERK4-mediated regulation of BR-independent cell death (Albrecht et al., 2008; Ma et al., 2016). Since SERKs also act as PTI amplifiers and as integration points for hormone-signaling networks (Heese et al., 2007; Roux et al., 2011; Nolan et al., 2017), similar mechanisms of redundancy and specialization are likely to operate during antiviral immune responses. Recent literature corroborates the notion that LRR-RLK coreceptors, including SERKs, function as modulators of developmental and immunity pathways (Heinlein, 2025).

### 3.2 The multifunctional coreceptor BAK1: a key node in LRR-RLK subfamily II and immune signaling

**Figure 5:**
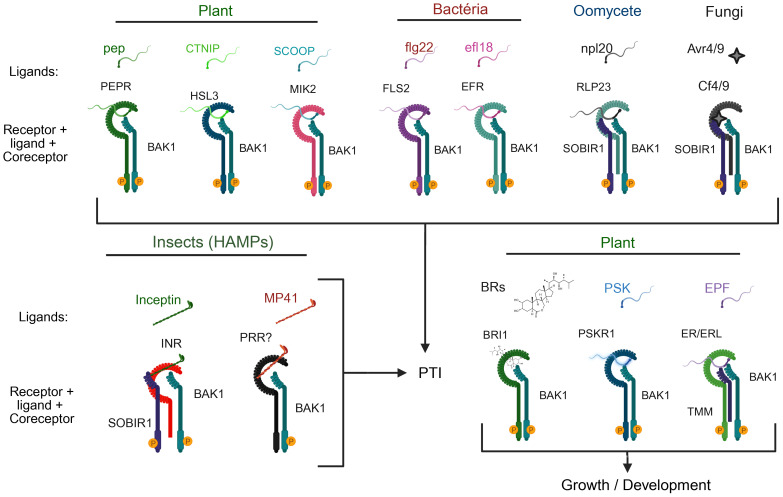
Versatile and flexible roles of BAK1 as a coreceptor in plant immune and growth signaling. BAK1 interacts with a variety of receptors involved in detecting damage-associated molecular patterns (DAMPs), pathogen-associated molecular patterns (PAMPs), and hormonal signals, contributing to immunity and growth regulation. Top: BAK1 forms associations with receptors involved in pathogen defense. For example, in plants, PEPR, HSL3, and MIK2 recognize the DAMPs Pep, CTNIP, and SCOOP, respectively, triggering PTI. In bacterial recognition, BAK1 serves as coreceptor for FLS2 and EFR, detecting flg22 and elf18, respectively. When sensing oomycete and fungal threats, BAK1 interacts with RLP23 and SOBIR1 to identify nlp20, or with Cf4/9 and SOBIR1 to recognize Avr4/9. Bottom left section: BAK1 contributes to herbivore-related molecular pattern (HAMP)-induced immunity by interacting with INR/SOBIR1 and an unidentified pattern recognition receptor (PRR)? to detect inceptin and MP41, respectively. Bottom right section: In the developmental context, BAK1 functions as a coreceptor for BRI1 (brassinosteroid receptor), PSKR1 (phytosulfocin sensor), and ER/ERL1 (with TMM) to regulate growth and development. These complexes highlight the flexible role of BAK1 as a coreceptor connecting different signaling pathways that govern pattern-triggered immunity (PTI) and growth/developmental responses in plants.

As a central member of the LRR-RLK subfamily II, BAK1 (or SERK3) is one of the most extensively studied coreceptors in plants. BAK1 was initially identified as a signaling coreceptor of the BR receptor BRI1 (Li et al., 2002; Nam and Li, 2002), but has since been recognized for its multiple regulatory functions that extend beyond growth, integrating signals from various developmental and immune pathways (**Figure 5**).

BAK1 displays the tripartite receptor configuration of the LRRII-RLK subfamily members, containing typically the short LRR ectodomain, a transmembrane segment, and a cytoplasmic kinase domain, which enables its rapid recruitment by ligand-bound receptors (**Figure 1**) (Chinchilla et al., 2009). As a universal coreceptor on the plasma membrane, BAK1 is capable of interacting with a variety of receptors, including RLKs and receptor-like proteins (RLPs) (**Figure 5**) (Ahmed et al., 2018; Albert et al., 2020; Wu et al., 2025a). This biochemical property is the foundation of its central role in balancing growth and defense signals across plant tissues (Liebrand et al., 2014; Yasuda et al., 2017).

BAK1 is a key component of the immune system and plays a significant role in initiating PTI by interacting with PRRs (**Figure 5**). To detect and transduce signals induced by endogenous damage-associated molecular patterns (DAMPs), the peptide (PEP) is recognized by PEP RECEPTOR (PEPR) 1 and 2, which interacts with BAK1 (Krol et al., 2010; Schulze et al., 2010). Upon perception of the stress peptide CTNIP, HAESA-LIKE 3 (HSL3) interacts with BAK1 (Rhodes et al., 2022). SERINE-RICH ENDOGENOUS PEPTIDE (SCOOP), another *Brassicacea* DAMP, is recognized by MALE DISCOVER 1-INTERACTING RECEPTOR-LIKE KINASE 2 (MIK2) driving its dimerization with BAK1 (Hou et al., 2021). The DAMP-induced cognate PRR-BAK1 complex formation activates signal cascade to initiate a typical PTI response.

BAK1 also interacts with FLS2 and EF-TU RECEPTOR (EFR), which are sensing receptors for bacterial PAMPs such as flagellin (or its active peptide flg22) and EF-TU (or its active peptide elf18) (**Figure 5**) (Chinchilla et al., 2007; Heese et al., 2007; Sun et al., 2013). Upon perception of the bacterial PAMP, FLS2 or EFR dimerizes with BAK1 and activates PTI through MAPK activation and the generation of ROS bursts. Additionally, BAK1 forms complexes with RECEPTOR-LIKE PROTEIN 23 (RLP23) and SUPPRESSOR OF BIR1-1 (SOBIR1) in response to necrosis-inducing peptides, such as nlp20 (Albert et al., 2015). Similarly, in tomato, BAK1 and SOBIR1 form an elicitor Avr4/9-induced complex with CF4/9, resulting in PTI signaling (Postma et al., 2016).

Interestingly, BAK1 contributes to defense against herbivory. In *Nicotiana attenuata*, NaBAK1 silencing reduces jasmonate accumulation and weakens defense against *Manduca sexta* (Yang et al., 2011). In *Nicotiana tabacum*, the INCEPTIN RECEPTOR (INR) mediates recognition of herbivore-associated molecular patterns (HAMPs) in a BAK1- and SOBIR1-dependent manner (Steinbrenner et al., 2020). BAK1 also serves as a coreceptor of a still unknown PRR that putatively recognizes MP41, a HAMP present in the saliva of a the small brown planthopper (BBPH) *Laodelphax striatellus* that is a BAK1-dependent PTI elicitor in several plant species (Qi et al., 2025). The role of BAK1 in immune responses to herbivory further underscores its multiple functions in plant immunity.

Furthermore, BAK1 serves as coreceptor in growth-related signaling pathways. In addition to the BR-induced BRI1-BAK1 activated complex for developmental signaling, BAK1 interacts with PHYTOSULFOKINE RECEPTOR (PSKR1) and CYCLIC NUCLEOTIDE-GATED ION CHANNEL 17 (CNGC17) to control cell expansion through the activation of proton pumps (Ladwig et al., 2015) and with ERECTA/ERL1 to regulate stomatal patterning and epidermal development (Lee et al., 2012; Jordá et al., 2016). In root tissues, BAK1 functions as a coreceptor for the FERONIA receptor, which recognizes RALF1, thus influencing cell expansion by repressing BR signaling (Dressano et al., 2017).

BAK1 is also involved in chitin-triggered immunity through its interaction with CHITIN ELICITOR RECEPTOR KINASE 1 (CERK1) (**Figure 3**) (Gong et al., 2019). Bacteria-induced BAK1 activation triggers the phosphorylation of CERK1 within the intracellular juxtamembrane region, priming the host for potential fungal attacks. The fungal-induced PTI is mediated by the CERK1:LYSM DOMAIN RECEPTOR-LIKE KINASE 5 (LYK5) complex, which recognizes the fungal PAMP chitin (Miya et al., 2007). This crosstalk mechanism between bacterial and fungal immunity, further demonstrates the ability of BAK1 to enhance multiple immune pathways simultaneously. Like NIK1 that is phosphorylated by BAK1, CERK1 serves as a substrate for activated BAK1, but, unlike NIK1, dissociates from the BAK1-PRR immune complex upon phosphorylation (Gong et al., 2019; Li et al., 2019a). However, it is still unknown whether other pathogen elicitors that activate BAK1, NIK1, or other LRRII-RLKs also modulate the PTI immune responses. Therefore, further investigation of these mechanisms may contribute to our understanding of the complex interplay between immune responses elicited by pathogens from different kingdoms.

Mechanistically, BAK1 complex formation depends on a ligand-dependent receptor and requires reciprocal transphosphorylation for signaling initiation (Bender and Zipfel, 2023). Specific phosphocodes within the kinase domain confer interaction specificity and define downstream outcomes, making BAK1 signaling highly modular and context-dependent (Perraki et al., 2018). Altogether, BAK1 exemplifies the functional plasticity of LRR-RLK subfamily II coreceptors, integrating developmental and immune cues across diverse signaling contexts. Its capacity to form dynamic complexes with a wide array of receptors underlies its central role in plant immunity, development, and adaptation.

Importantly, emerging studies suggest that BAK1 is also involved in antiviral responses, both directly and indirectly. For example, viral manipulation of BAK1-mediated pathways has been observed, and the formation or suppression of BAK1-containing complexes can influence the outcome of viral infections (Kørner et al., 2013; Huang et al., 2023; Robinson et al., 2025). These insights open new avenues for exploring how viruses exploit or evade BAK1-dependent immune signaling.

### 3.3 Negative regulation of BAK1 activity

As a universal coreceptor, the complex formation between BAK1 and ligand-activated receptors must be precisely regulated to prevent overactivation in the absence of stimuli. As described in item 2.2, members of the LRR-RLK II subfamily, including NIK1 and NIK2, negatively modulate the formation and activation of the FLS2/BAK1 complex (Li et al., 2019a). Additionally, the BIR-type receptors (BAK1-INTERACTING RECEPTOR-LIKE KINASES) are central negative regulators of BAK1 activity (Halter et al., 2014; Imkampe et al., 2017).

**Figure 6:**
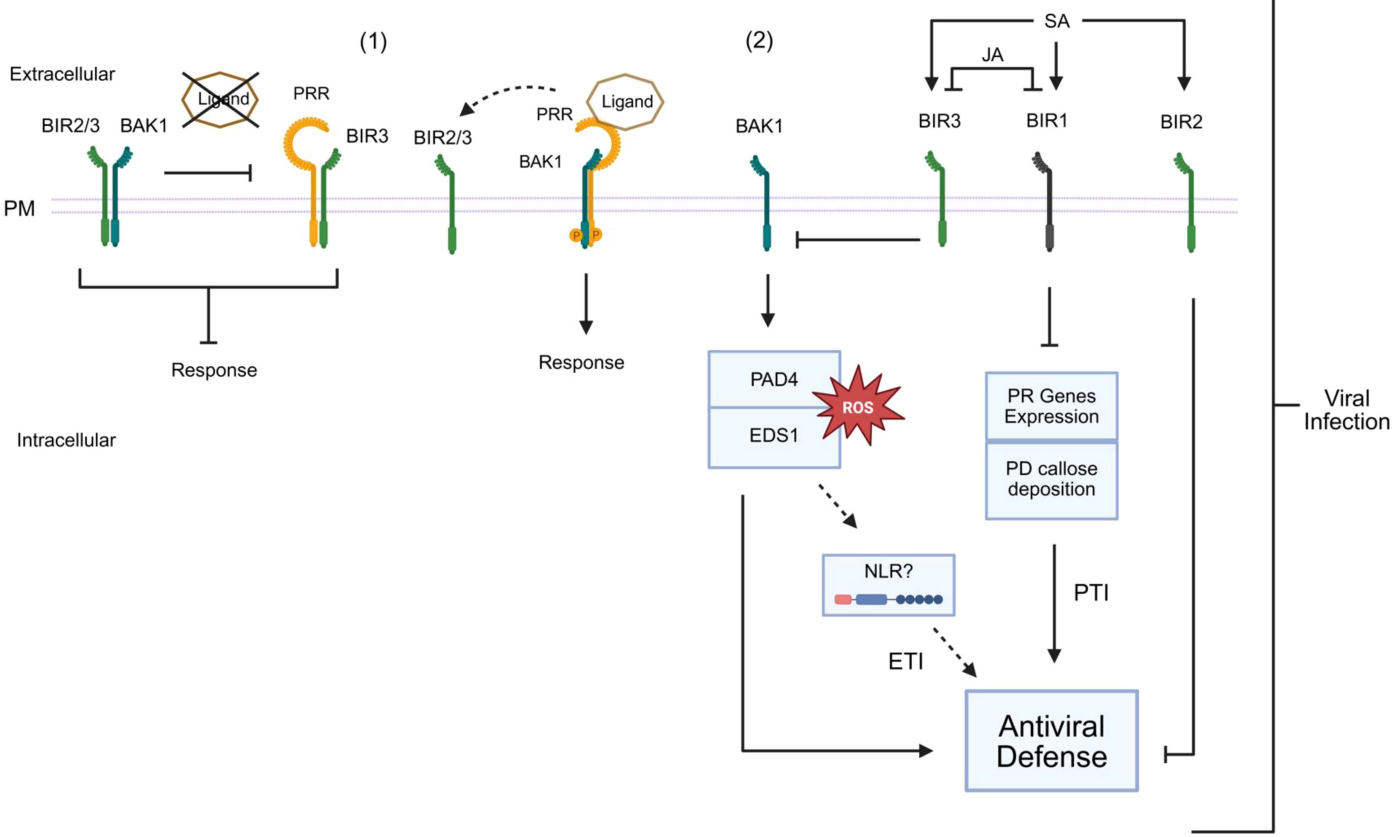
BIR negatively modulates BAK1 activation and antiviral responses. (**1**) BIRs function as central negative regulators of BAK1 activity. BIRs contain intracellular pseudokinase domains and exert their regulatory role primarily by sequestering BAK1 in inactive complexes. In the presence of the ligand, PRR recruits BAK1 forming and activated immune complex, releasing BIR2/3. (**2**) BIR-mediated regulation of antiviral defense. BIR1 and BIR3 are induced during viral infection, influenced by antagonistic interactions between salicylic acid (SA) and jasmonic acid (JA) signaling. BIR2 induction during infection is affected by SA, but not by JA. BIR1 negatively regulates antiviral defense through mechanisms that may include PTI gene expression and plasmodesmata (PD) callose deposition, as well as unidentified pathways independent of reactive oxygen species (ROS) or the signaling components BAK1, SOBIR1, or PAD4. BIR3 represses an antiviral response, which requires BAK1- and EDS1/PAD4-dependent activation of effector-triggered immunity (ETI), likely involving intracellular nucleotide-binding leucine-rich repeat receptors (NLRs), leading to asymptomatic resistance. Solid arrows indicate activation, blunt arrows denote repression, and dashed arrows represent potential effects on antiviral defenses.

BIR2 and BIR3, belonging to the LRR-X subfamily, were initially identified as interactors of BAK1 by immunoprecipitation coupled with mass spectrometry (Halter et al., 2014; Imkampe et al., 2017). Unlike BAK1, which possesses a functional kinase domain, BIRs contain intracellular pseudokinases and exert their regulatory function primarily by sequestering BAK1 into inactive complexes (**Figure 6**). Crystallographic structures have revealed that the extracellular domains of BIRs interact with critical regions of BAK1 and SERK1, which are required for recruitment by activated receptors, thereby preventing the formation of the signaling complex (Ma et al., 2016; Hohmann et al., 2017). The activation of PTI or BR signaling requires that a ligand-activated receptor, such as FLS2 or BRI1, competes successfully with BIRs for binding to BAK1 (**Figure 6**). This reversible competitive inhibition mechanism, a crucial control point, ensures that downstream signaling occurs only under appropriate physiological conditions, both in immunity and in response to BR. The negative role of BIR3 not only depends on BAK1 but also on the ENHANCED DISEASE SUSCEPTIBILITY 1 and its coreceptor PHYTOALEXIN DEFICIENT4 (EDS1/PAD4) complex, suggesting interference with intracellular nucleotide-binding leucine-rich repeat receptor (NLR)-type receptor signaling (**Figure 6**). BIR2 has also been identified as a repressor of tobacco rattle virus (TRV) resistance, reinforcing the idea that BIR family members play a conserved and crucial role as negative modulators of antiviral immunity (**Figure 6**). During TRV infection, the *BIR1* and *BIR3* expression is antagonistically modulated by the salicylic acid (SA) and jasmonic acid (JA) signaling pathways, respectively, indicating a complex interplay between these immune components (Robinson et al., 2025).

In contrast, BIR1 plays a significant role in suppressing antiviral defense through mechanisms that include modulation of genes associated with PTI and increased callose deposition in plasmodesmata, independently of BAK1-, SOBIR1-, or PAD4-mediated pathways. During infection, BIR1 is involved in a homeostatic mechanism, which balances immune activation and prevents excessive damage to the host (Robinson et al., 2025).

BAK1-dependent PTI responses are also modulated by the IMPAIRED OOMYCETE SUSCEPTIBILITY1 (IOS1), a malectin-like/LRR-RLK that is a critical PTI player (Yeh et al., 2016). On the plasma membrane, IOS1 associates with BAK1-dependent PRRs, FLS2, and EFR. IOS also associates with BAK1 independently of the ligand and positively regulates the formation of the FLS2 or EFR and BAK1 complex upon recognition of bacterial PAMPs.

### 3.4 SERKs 1/3/4 and their emerging role in antiviral immunity

RNA silencing has been considered the primary defense against viruses in plants (Lopez-Gomollon and Baulcombe, 2022). However, several lines of evidence have demonstrated that viral infections also trigger canonical immune responses. These include MAPK activation, increased SA and ethylene production, callose deposition, or upregulation of PTI-related genes (Kørner et al., 2013; Nicaise, 2014; Calil and Fontes, 2017).

The BAK1(SERK3) coreceptor has been widely recognized for its role in classical immunity pathways against non-viral pathogens. Emerging evidence also reveals a role for BAK1 in antiviral immunity. The *bak1* and *bak1bkk1* (also designated BAK1-LIKE KINASE 1) silenced lines exhibit increased susceptibility to infection by several RNA viruses, including turnip shrivel virus (TCV), tobacco mosaic virus (TMV), oilseed rape mosaic virus (ORMV), plum pox virus (PPV), and TRV (Yang et al., 2010; Kørner et al., 2013; Robinson et al., 2025). This susceptibility phenotype suggests that BAK1 acts as a positive regulator of antiviral immunity, possibly integrating viral PTI and effector-triggered immunity (ETI) (Niehl et al., 2016; Huang et al., 2023). Accordingly, viral infections trigger the activation of typical PTI responses, including ROS accumulation, defense gene upregulation (such as PR-1), SA accumulation, and callose deposition (Nicaise, 2014). It is worth mentioning that BAK1 (SERK3), as an interactor of NIK1 and an activator of the antiviral pathway, may also be associated with reduced viral load and attenuated symptoms in plants (**Figure 3**) (Li et al., 2019a).

**Figure 7:**
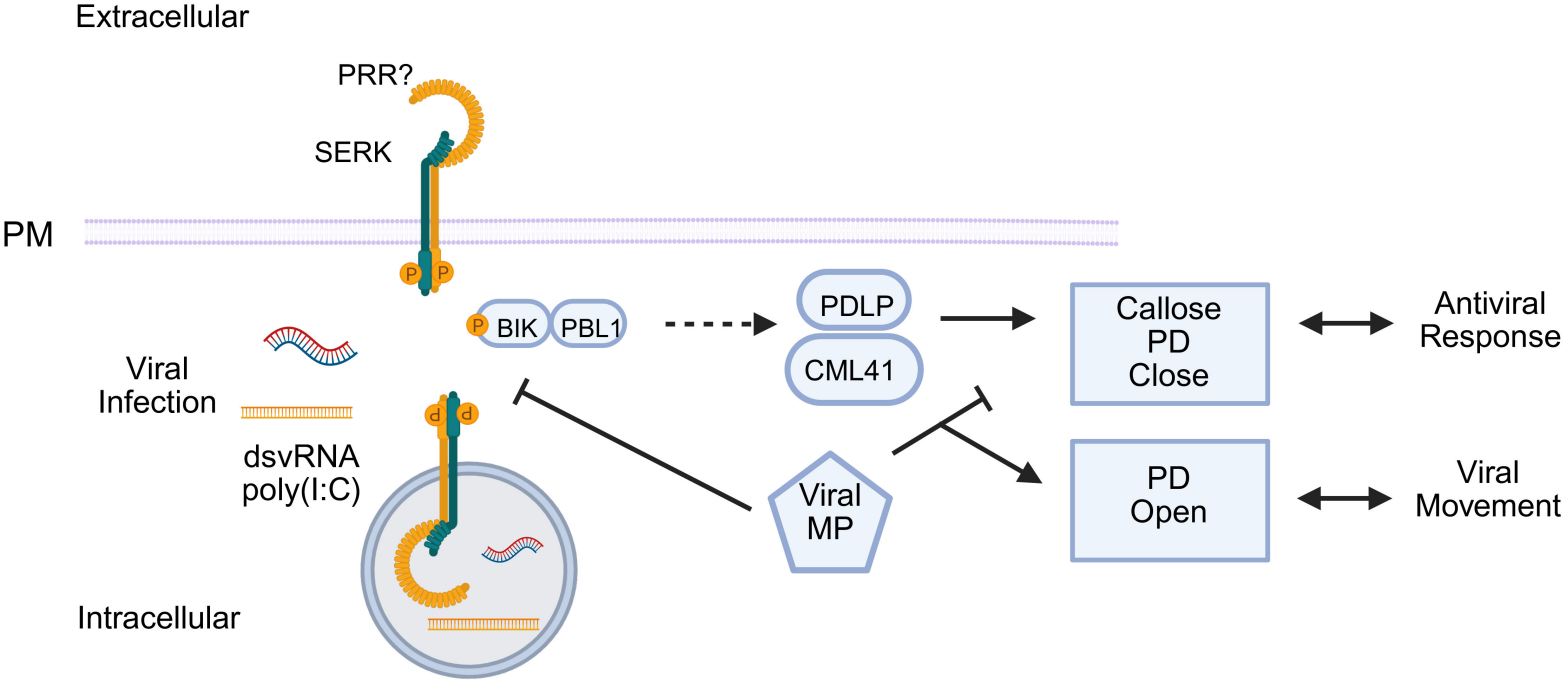
SERK1 responds to dsRNA/poly (I:C), but viruses block the response. SERK1 triggers PTI-like signaling through BIK/PBL1 and PDLP/CML41, leading to Ca^2+^ influx, callose deposition, and plasmodesmata (PD) closure, thereby hindering viral movement. As a counter-defensive measure, the viral movement proteins (MP) suppress this pathway, promoting the increase in the plasmodesmata exclusion limit to allow cell-to-cell virus movement.

In addition to its well-established role in plant tissue development, the coreceptor kinase SERK1 is also involved in the immune response to viral infections. Experimental evidence indicates that treatment with the double-stranded viral RNA analog polyinosinic:polycytidylic acid [poly(I:C)] activates the MAP kinase signaling cascades involving MPK3 and MPK6. As ethylene biosynthesis is a typical marker of PTI activation, *serk1* mutant plants display a significant impairment of ethylene production in response to poly(I:C). Furthermore, the *serk1* mutants do not exhibit substantial antiviral resistance when pre-treated with poly(I:C) before viral inoculation, suggesting that SERK1 is essential for the effective induction of immune responses mediated by the recognition of exogenous viral RNA (Niehl et al., 2016). Recent progress in elucidating a layer of the dsRNA-mediated antiviral response includes the identification of intermediate signaling components and the nature of the antiviral host defense (Huang et al., 2023; Heinlein, 2025). These include SERK1 along with the BOTRYTIS INDUCED KINASE1 (BIK1), PLASMODESMATA-LOCATED PROTEINs (PDLP) 1/2/3, as well as CALMODULIN-LIKE 41 (CML41) and Ca²⁺ signals. Once activated, the dsRNA-induced antiviral immunity induces callose deposition at the plasmodesmata, restricting viral movement. T counteract this defense mechanism, the movement proteins of distinct RNA viruses suppress the dsRNA-mediated immunity, decreasing callose deposition at the plasmodesmata and allowing the cell-to-cell movement of viral RNA (**Figure 7**). However, the primary receptor responsible for dsRNA recognition remains unidentified.

BAK1 (SERK3), on the other hand, is involved in modulating the BIR3-mediated response, which negatively regulates antiviral resistance in a BAK1- and EDS1/PAD4-dependent manner, characterizing a pathway similar to ETI (Robinson et al., 2025). In contrast, BIR1, as a repressor of antiviral resistance, inhibits PTI-associated gene expression and callose deposition, independently of BAK1. These findings advance our understanding of the role of SERKs as an integrator of multiple immune signals, positioning them as a key component at the interface between numerous stresses.

## 4 CIKs: molecular regulation of meristematic homeostasis and involvement in plant immunity

NIK3 (or CIK1) was also first identified through its interaction with the begomovirus NSP, and the loss of NIK3 function in knockout lines implicated this coreceptor in antiviral immunity (Fontes et al., 2004). Later, NIK3 was identified as a relevant hub in the cell surface network of protein-protein interactions, which clustered the NIK1 and NIK2 hubs together and separated from the NIK3 hub (Ahmed et al., 2018; Smakowska-Luzan et al., 2018; Li et al., 2019a). Therefore, it is not surprising that NIK3, later designated CIK1, has functionally diverged from NIKs, particularly in development (Sakamoto et al., 2012; Li et al., 2019a; Zhu et al., 2021).

The root apical meristem (RAM) and shoot apical meristem (SAM) maintain self-renewal while producing differentiated cells that form various organs (Byrne et al., 2003). The CIK clade, including CIK1 (NIK3), CIK2, CIK3, and CIK4, belongs to the LRR-RLK subfamily II of coreceptors and is essential for SAM and RAM maintenance (Hu et al., 2018). In studies on receptors that participate in meristem homeostasis, the genes AT5G16000 and AT3G25560, which are members of the LRR-RLKII subfamily, were designated as CIK5 and CIK6, respectively (Cui et al., 2018; Hu et al., 2018; Zhu et al., 2021). However, CIK5 and CIK6 were previously designated as NIK1 and NIK2, respectively, for their functions in antiviral immunity in plants (Fontes et al., 2004; Zorzatto et al., 2015; Li et al., 2019a). In this review, the role of NIK1/2 has been discussed in antiviral immunity and as a signaling hub that serves the same signaling circuit to relay biotic and abiotic stress into a shared physiological response.

This balance between stem cell maintenance and differentiation is governed by conserved signaling pathways, the CLAVATA (CLV) pathway (Carles and Fletcher, 2003; Gross-Hardt and Laux, 2003). CLV1, CLV2, CLV3, WUSCHEL (WUS), and the BARELY ANY MERISTEM (BAM1/2) proteins are essential key regulators that operate to maintain meristem homeostasis (Nimchuk et al., 2015). The transcription factor WUS, expressed in the organizing center of SAM, promotes stem cell identity and induces CLV3 expression (Perales et al., 2016). CLV3 encodes a CLV3/EMBRYO SURROUNDING REGION (CLE) peptide recognized by receptors such as CLV1, a leucine-rich repeat receptor-like kinase (LRR-RLK), which triggers downstream differentiation signals (Clark et al., 1997; Fletcher et al., 1999). As a negative feedback loop, CLV3 activates CLV1 to suppress WUS expression and maintain meristem size homeostasis (Brand et al., 2000; Schoof et al., 2000). CLV2, an LRR-RLP lacking kinase activity, functions in conjunction with the pseudokinase CORYNE (CRN) to form a receptor complex that operates in parallel to CLV1, contributing to meristem regulation and reproductive organ development (Kayes and Clark, 1998; Jeong et al., 1999; Agarwal et al., 2022; Bashyal et al., 2024).

The CIKs proteins have the canonical structure of subfamily II members and operate as coreceptors with CLV1/2 and RECEPTOR-LIKE PROTEIN KINASE 2 (RPK2) in SAM (Hu et al., 2018; Zhu et al., 2021) and with ARABIDOPSIS CRINKLY4 (ACR4) and RECEPTOR-LIKE KINASE 7 (RLK7) in RAM to modulate stem cell signaling (Hu et al., 2022; Meng et al., 2025). In RAM, CIKs also serve as coreceptors for ACR4 in the CLE40 pathway, maintaining distal meristem organization through ligand-independent interaction with ACR4 (Hu et al., 2022; Suresh et al., 2025). As key regulators in lateral root initiation and spacing in *Arabidopsis thaliana*, they directly interact with the RLK7 receptor to be phosphorylated upon treatment with the TOLS2 peptide, a ligand known to suppress lateral root initiation. The TOLS2–RLK7–CIKs signaling pathway relay information to the MKK4/5–MPK3/6 cascade to regulate the expression of the transcription factor PUCHI, essential for the spatial control of cell division during lateral root formation (Meng et al., 2025).

While CIK clade members exhibit functional redundancy in SAM, recent studies have revealed their distinct regulatory effects in RAM (Zhu et al., 2021), underscoring their significant role of CIKs in the CLE40–ACR4–WOX5 signaling pathway. This highlights their conserved yet distinct functions in different plant meristems, providing insights into the molecular mechanisms that govern stem cell maintenance in plants (Cui et al., 2018; Hu et al., 2022; Zhu et al., 2023; Meng et al., 2025). Furthermore, CIKs also associate with BAM1/2 and RPK2 to control somatic cell fate determination during early anther development in Arabidopsis (Cui et al., 2018).

The BAM1/2/3 receptors, structurally similar to CLV1, exhibit opposite activity, promoting stem cell proliferation. The triple mutant exhibits severely reduced meristems, indicating that BAMs respond to distinct CLE peptides and enhance meristem robustness (DeYoung et al., 2006; DeYoung and Clark, 2008). Recent work has demonstrated that CIK1 functions as a coreceptor of BAM1/2 and RPK2, playing a crucial role in regulating archesporial cell division and parietal layer specification during early anther development (Cui et al., 2022).

The *cik1/2/3/4/5/6* mutants exhibit the same phenotype as *bam1/2* and *rpk2*, displaying significantly smaller rosettes and leaves, dramatically more rosette leaves, and a significantly enlarged and irregular SAM. Moreover, these exhibit abnormal anticlinal divisions and defective anther wall organization, highlighting the overlapping roles of these pathways. Although CIKs function redundantly, they contribute unequally to regulate stem cell homeostasis. CIK1 is the most critical CIK in this process (Hu et al., 2018; Zhu et al., 2021). In contrast, the involvement of CIK1 (NIK3) in plant defenses remains a matter of debate. Reverse genetics assays not only implicate NIK3 as an antiviral receptor but also as a negative regulator of ETI and PTI (Fontes et al., 2004; Ahmed et al., 2018). Accordingly, the structural and functional overlap of CIKs with other members of subfamily II of LRR-RLKs, particularly NIKs, suggests their potential involvement in broader signaling networks that mediate the interplay between development and defense. Further mechanistic studies, particularly those investigating pathogen-specific responses involving CIK1/NIK3, are needed to fully elucidate the extent of their regulatory functions in plant immunity.

## 5 LRRII-RLK coreceptors linking development, meristems, and plant-virus interactions

Viruses have developed strategies to ensure their survival and transmission (Garzo et al., 2020). They can alter both the behavior of insect vectors and the secondary metabolites produced by the plants to improve their attractiveness (Yu et al., 2024). At the other side, plants fed by insect vectors can affect the viral life cycle, genetic population, and evolution of the viruses (Gutiérrez et al., 2013). Plant viruses have coevolved to exploit the spatial and molecular complexity of the host tissues. The ability of viruses to manipulate and coevolve in contact with a dynamic signaling network highly enriched in LRRII-RLKs may be a key link in understanding the biology behind plant-virus interactions. For instance, early meristematic regions, such as SAM and RAM, represent developmental niches to which plant growth and immunity signaling pathways converge. Meristems exhibit high activity of relevant coreceptors, including CIKs, SERKs, and NIKs, as can be seen in the ePlant tool and database (https://bar.utoronto.ca/eplant/), which shows high transcription levels of CIKs, SERKs, and NIKs in meristematic tissues, essential for the direct or indirect maintenance of growth and defense homeostasis (Li et al., 2024; Xu et al., 2025).

Meristematic tissues are often virus-free or display limited viral infection due to a combination of antiviral mechanisms, including RNA interference (RNAi), post-transcriptional and transcriptional gene silencing (PTGS and TGS), and RNAi-independent defenses such as WUS-mediated regulation and plasmodesmata closure (Bradamante et al., 2021; Incarbone et al., 2023). The mechanisms involved with RNAi have also been well reviewed (Li and Wang, 2019; Meier et al., 2019; Guo et al., 2020; Jin et al., 2021; Venu et al., 2025). The other RNAi-independent mechanisms have a link with LRRII-RLK members and are discussed here.

The transcription factor WUS, which is crucial for maintaining stem cell identity in the SAM, has been involved in LRRII-RLKs-dependent and RNAi-independent antiviral defense. The stem cell regulator WUS responds to CMV infection and represses virus accumulation in the central and peripheral zones of the meristem. Furthermore, WUS inhibits viral protein synthesis by repressing S-adenosyl-L-methionine-dependent methyltransferases, which are involved in ribosomal RNA processing and ribosome stability. Thus, plants employ a translation regulatory strategy to protect meristems against viruses, uncovering the underlying mechanism for WUS-mediated broad-spectrum innate antiviral immunity (Wu et al., 2020). CLV modulates WUS expression through the CIK signaling pathway, placing this developmental module at the interface of growth and immunity (Hu et al., 2018).

Furthermore, WUS plays a role in the cytokinin (CK) hormone response (Xie et al., 2018), which appears to have a contradictory role in plant antiviral immunity. In contrast to the CKs produced by many biotrophic bacterial and fungal pathogens to facilitate their proliferation in host plants, plant-derived CKs may play a role in enhancing plant resistance to viral infection (Sano et al., 1994; Masuta et al., 1995; Sano et al., 1996). However, the molecular mechanisms of CK-mediated resistance to viral infections remain unclear (Choi et al., 2011). Given the different reactions of CK to viruses and an emerging but limited understanding of CKs in plant defense (Akhtar et al., 2020; Zhao and Li, 2021), research on the role of CKs in tripartite interactions (plant, vector, virus) also appears to be timely (Pan et al., 2021).

The antagonism between cytokinin and auxin is well-established in controlling plant growth/development, and is essential for determining morphogenetic patterns and the dynamics of cell differentiation (Su et al., 2011; Kurepa and Smalle, 2022). Although traditionally associated with development, evidence suggests that this hormonal interaction also plays a central role in regulating plant immunity and signaling networks associated with defense against pathogens (Naseem and Dandekar, 2012). Notably, auxin has been shown to directly regulate the subcellular localization of coreceptors from the LRRII-RLK subfamily, including CIK2, CIK3, and CIK5. A maximum auxin gradient in pericycle cells induces the vacuolar internalization of CIKs, thereby preventing the formation of the TOLS2–RLK7 receptor complex and promoting cell division activation during the determination of lateral root founder cells (Meng et al., 2025). These auxin-dependent mechanisms, which regulate the subcellular localization of CIKs and integrate hormonal signaling with the subcellular distribution of coreceptors, may represent strategic targets at the interface between development and immunity, potentially exploited by pathogens to modulate defense responses in plants.

A crucial mechanism is the control of plasmodesmata opening, which is essential for the cell-to-cell movement of viruses and is tightly regulated in meristematic tissues. This control involves receptors of the BAM family, whose signaling is mediated, in various contexts, by coreceptors of the LRR-RLK II subfamily, such as CIKs. The viral protein C4, encoded by geminiviruses and widely characterized for its role as a suppressor of gene silencing, interacts directly with host proteins (Fontes et al., 2021). Independent studies have demonstrated that C4 from viruses such as tomato yellow leaf curl virus (TYLCV) and mungbean yellow mosaic virus (MYMV) physically interacts with the receptors BAM1 and BAM2, members of the LRR-RLK XI subfamily, inhibiting their function in the intercellular propagation of RNA-interfering (RNAi) signals (Carluccio et al., 2018; Rosas-Diaz et al., 2018). Although BAM1 and BAM2 are traditionally associated with shoot meristem maintenance, they also play a relevant role in facilitating the movement of RNAi from vascular tissues. Interaction with the C4 protein disrupts this process, favoring the establishment of systemic viral infection. Interestingly, this viral interference does not compromise other physiological functions of BAM1/2 related to development. The exact mechanism by which BAM1/2 facilitates RNAi transport remains poorly understood. As signaling partners of BAMs in cellular differentiation, a further examination of CIK coreceptors in the systemic movement of RNAi would be of extreme interest.

The potential role of CIKs in BAM1/2-mediated antiviral signaling is further supported by the functional analogy with other LRRII-RLKs in plasmodesmata functioning. In fact, members of the SERK family, particularly SERK1 and BAK1, have also been implicated in regulating plasmodesmata permeability and immune signaling during viral infections (Huang et al., 2023).

**Figure 8:**
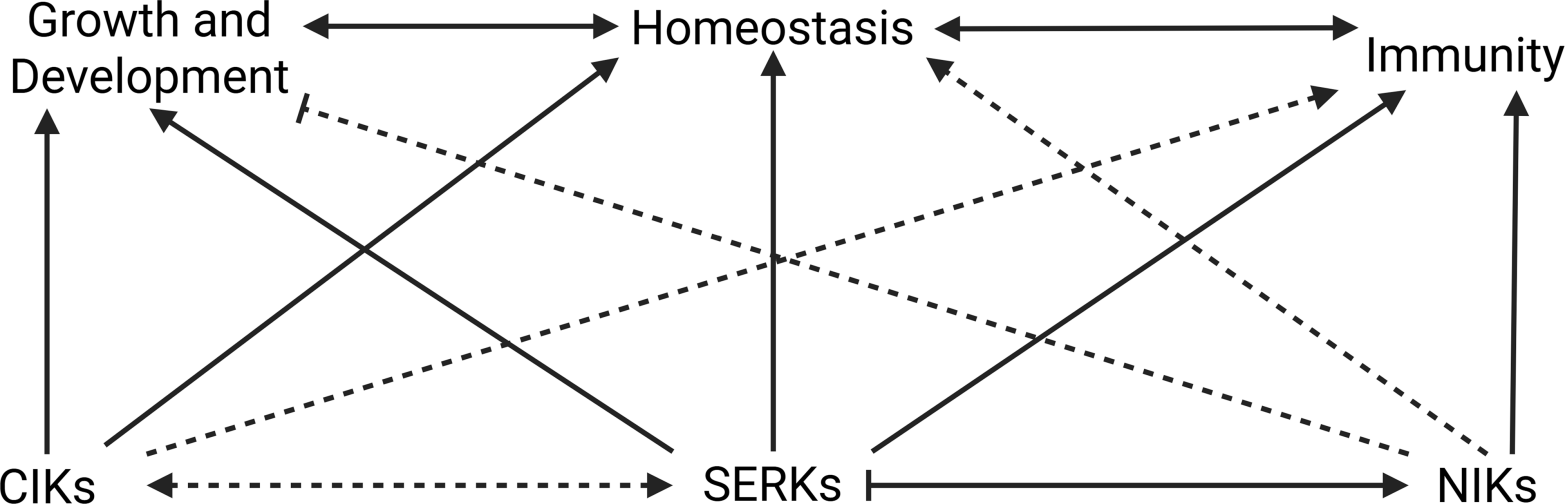
CIKs, SERKs, and NIKs participate in growth/development homeostasis and immunity in plants. Solid arrows represent relationships well described in the literature, and dashed arrows indicate relationships that require further investigation. SERKs are central coreceptors involved in plant growth/development, homeostasis, and immunity. Joint participation with CIKs has been little investigated, but SERKs interactions with NIKs has been reported. SERKs activates the NIK-mediated pathway, while the SERK-mediated pathway may be attenuated by NIKs. Research exploring NIKs’ role in growth and development is still emerging, but their participation in immunity has been characterized. CIKs are involved in growth, development and meristem homeostasis, but there is little direct information regarding their involvement in immunity. Their relationships with other LRRII-RLK members, SERKs and NIKs, have been little examined.

Furthermore, NIKs, as known for their interaction with the begomovirus NSP effector, function as antiviral kinases that repress ribosomal gene expression in response to viral, bacterial, drought, and osmotic stress. This repression leads to the inhibition of global translation and, consequently, of viral protein synthesis, while negatively regulating genes involved in photosynthesis, as a platform for crosstalk between the immune response and growth regulation (**Figure 8**) (Zorzatto et al., 2015; Ferreira et al., 2025). Collectively, these findings suggest that shoot and root meristems represent a convergent point of developmental control and antiviral defense, in which the coreceptors of the LRR-RLKII subfamily CIKs, SERKs, and NIKs play overlapping and context-dependent roles in integrating growth regulation with immune responses to maintain cellular homeostasis.

In summary, LRR-RLKII coreceptors, including CIKs, SERKs, and NIKs, may emerge not only as key regulators of growth and development but also as molecular targets of viral manipulation. For example, PTI-like activity against RNA viruses, mediated by SERK1, is inhibited by viral MP. Meanwhile, NIK1, a central antiviral kinase, is neutralized by NSP to suppress translational regulatory-based defenses and photosynthesis. CIKs, particularly active in meristems, coordinate signaling for stem cell maintenance, and their receptor BAM is a target of viral manipulation via C4. These may also represent points of homeostasis between viral infection and plant development/growth.

The functional integration of these coreceptors into hormonal, defense, and developmental programs highlights their role as signaling hubs at the molecular crossroads of plant physiology and pathogen attack (**Figure 8**). This coevolutionary triad is supported by various aspects: (i) meristematic tissues exhibit high LRRII-RLK activity and low viral infection activity, (ii) viruses have strategies that manipulate proteins, which participate in plant growth/development, just as several LRRII-RLK coreceptors, (iii) plants dynamically adjust coreceptor activity to balance growth and immunity, defining a highly specialized interaction landscape. Investigating these molecular relationships may reveal novel resistance strategies, particularly by modulating the expression or activity of LRR-RLKII members, such as CIKs, SERKs, and NIKs, in developmental niches critical for infection.

## 6 Examples of LRRII-RLKs and viral infection in other plant species

Like in Arabidopsis, the members of the tomato (*Solanum lycopersicum*) LRRII-RLK subfamily genes exhibit tissue-specific expression patterns, particularly in young organs such as developing leaves and cotyledons (Sakamoto et al., 2012). Recent studies in sugarcane (*Saccharum* sp*ontaneum)* identified 27 LRRII-RLK genes, with a notable expansion in the LRRII-C clade (9 genes), compared to only two in sorghum and three in rice. This expansion, likely resulting from polyploidy, correlates with differential responses to pathogens such as sugarcane mosaic virus (SCMV) and the fungus *Sporisorium scitamineum.* For example, SsLRRII-RLK4 in the SERK clade is strongly induced during SCMV infection, while SsLRRII-RLK3–2 in the NIK clade shows complex regulation during fungal interaction (Ding et al., 2025). Within the LRR-RLKII subfamily, the LRRII2c clade shows potential direct involvement in pathogen responses.

An example of the functional versatility of LRRII members is their involvement in virus-induced immune suppression. In *Crataegus pinnatifida* (hawthorn), the apple chlorotic leaf spot virus (ACLSV) produces a virus-derived small interfering RNA, named vsiR1360, which targets the *CpLRR-RLK1* gene, a member of the LRRII-C subfamily (Guo et al., 2020). This vsiRNA binds to the 5′ untranslated region (5′UTR) of *CpLRR-RLK1* mRNA, recruiting the RNA-induced silencing complex (RISC) and promoting mRNA degradation via PTGS. Functionally, *CpLRR-RLK1* acts as a positive regulator of basal immunity by promoting ROS accumulation, callose deposition, and the expression of defense-related genes such as *FRK1* and *WRKY29*, resembling a canonical PTI response. Virus (vsiR1360)-induced *CpLRR-RLK1* silencing impairs these immune responses and compromises resistance to *Pseudomonas syringae* pv. *tomato.* These results reveal a precise mechanism by which the virus hijacks the host’s silencing machinery to suppress receptor kinase-mediated defense. In addition, they demonstrate that LRRII and LRRII2c are dynamic gene families, in which structural conservation enables functional diversification, particularly in pathogen defense. Future research should prioritize identifying specific ligands, characterizing downstream signaling pathways, and investigating how polyploidy-driven gene duplication shapes plant immune resilience.

Recently, the membrane kinase BAK7, also known as SERK4 or BKK1, was identified as a key regulator of submergence tolerance in *Brassica napus* and *Arabidopsis thaliana*, through a mechanism involving the nuclear translocation of its cytoplasmic portion and interaction with the transcription factor TCP21. Phosphorylation and stabilization of TCP21 by SERK4 establishes a positive feedback loop, amplifying the adaptive response to hypoxic stress (Guo et al., 2025). Interestingly, TCP21 was also identified as a critical target of the ortho-tospovirus tomato spotted wilt virus (TSWV). The viral effector NSs hijacks TCP21 to interfere with hormone receptors COI1, TIR1, and MAX2, suppressing hormonal defense pathways in the plant *Capsicum chinense*. In this pathway, the NLR Tsw detects TCP21 manipulation by NSs and activates antiviral immunity (Chen et al., 2023). The functional connection between SERK4/BAK7 and TCP21, along with their central role in NLR-mediated antiviral surveillance, suggests that the BAK7–TCP21 pathway may serve as an interface sensitive to biotic and abiotic stresses, potentially modulating antiviral immunity in plants. Although the BAK7 study has not focused on viral immunity, its results open a promising perspective for future investigations in this context.

Recent research in rice (*Oryza sativa*) has unveiled a sophisticated viral strategy employed by the rice grassy stunt virus (RGSV) to suppress host antiviral immunity by hijacking a developmental coreceptor kinase (Wu et al., 2025b). Under normal conditions, SERK4 expression is tightly regulated to balance growth and defense. However, upon RGSV infection, the viral protein P3, functioning as a transcription activator-like effector (vTALE), binds directly to the promoter of *SERK4*, leading to a strong upregulation of *SERK4*, which in turn phosphorylates the E3 ubiquitin ligase P3IP1. The activated P3IP1 targets NRPD1a, a core subunit of RNA polymerase IV, which is essential for the RNA-directed DNA methylation (RdDM) antiviral pathway, for proteasome-mediated degradation. The degradation of NRPD1a impairs the biogenesis of siRNAs, ultimately weakening antiviral defenses and enabling systemic virus proliferation. Interestingly, transgenic and mutant rice lines confirmed that suppression of SERK4 or its downstream partners (P3IP1 and NRPD1a) can mitigate viral susceptibility and developmental defects (Wu et al., 2025b). These findings position SERK4 as a convergence point between growth regulation and immunity and reveal a rare case in which a viral protein mimics plant transcriptional machinery to rewire receptor kinase-mediated signaling, reinforcing the relevance of LRR-RLKII members such as SERKs beyond Arabidopsis in plant-virus interactions.

Cassava mosaic disease (CMD), caused by begomoviruses such as south African cassava mosaic virus (SACMV), poses a critical threat to cassava production, especially in Africa. Genetic resistance to CMD is associated with loci such as CMD1, CMD2, and CMD3, although the underlying genes are not yet fully characterized. The tolerant variety TME3 stands out for its resilience, contrasting with the persistent susceptibility of T200. Studies demonstrate that TME3 tolerance is not based on a more robust initial immune response, but rather on the delayed activation of defense mechanisms mediated by ETI and SA, capable of suppressing viral virulence factors (Sizani et al., 2025). The temporal dynamics of viral gene expression, such as AV1 and AC2, reveal their critical role in the early stages of infection, while genes such as BV1 and AC4 become more highly expressed in the later stages. The recovery observed in TME3 coincides with a decrease in viral expression, especially of BV1, and with the upregulation of defense components, including WRKYs and the kinase NIK3. The latter was shown, by comparison between genotypes, to be a possible functional antagonist of NIK1 and NIK2, whose expression is associated with susceptibility in cassava (Sizani et al., 2025). These findings suggest that CMD tolerance in cassava does not depend on preventing the initial infection, but on the ability of the plant to reconfigure its immunity over time to effectively suppress viral replication. Since the molecular battle between the plant and virus evolves throughout the infection, viral effectors may be more efficient at the beginning of the infection than at later stages. Further investigation is needed to elucidate this mechanism. Collectively, these findings demonstrate that LRRII-RLK genes involved in antiviral immunity occur in various economically relevant plant species. A summary of LRRII-RLKs orthologs associated with antiviral immunity in different crops is provided in **Table 1**.

**Table 1:** Examples of LRR-RLK receptors of subfamily II associated with antiviral immunity in crops and non-model plants such as sugarcane, hawthorn, pepper, rice, and cassava.

Plant species	Virus	Disease	Description	Reference
*Saccharum* sp*ontaneum* (sugarcane)	sugarcane mosaic virus (SCMV)	Sugarcane mosaic	High level of expression of the SsLRRII-RLK gene during the SCMV viral infection suggests a possible role for this gene in the plant’s response to viral biotic stress.	(Ding et al., 2025)
*Crataegus pinnatifida* (hawthorn)	chlorotic leaf spot virus (ACLSV)	Apple chlorotic leaf spot	The ACLV virus produces a micro-RNA (vsiR1360), which targets the downregulation of CpLRR-RLK mRNA, recruiting the RISC complex and positively regulates the host’s basal immunity.	(Guo et al., 2020)
*Capsicum* *chinense* (pepper)	tomato spotted wilt orthotospovirus (TSWV)	Tomato spotted wilt	By targeting the TCP21 protein to inhibit phytohormone receptors, the pathogen’s effector has its virulence action detected by the NLR Tsw protein, activating antiviral immunity (Chen et al., 2023). Interestingly, TCP21 can interact with SERK4/BAK7 (Guo et al., 2025).	(Chen et al., 2023); (Guo et al., 2025).
*Oryza sativa* (rice)	rice grassy stunt virus (RGSV)	Rice grassy stunt	The P3 protein acts as a transcription factor encoded by the virus, which positively regulates SERK4 by phosphorylating P3IP1, leading to NRPD1 degradation and attenuating the antiviral defense based on RNA-directed DNA methylation (RdDM).	(Wu et al., 2025b)
*Manihot esculenta* Crantz (cassava)	South African cassava mosaic virus (SACMV)	Cassava mosaic disease	The resistant variety (TM3) activates a more robust antiviral immunity such as (ETI) and the Salicylic Acid (SA) pathway. This suppresses viral replication through the positive regulation of *WRKY* genes and the NIK3 protein kinase, in contrast to the susceptible variety (T200).	(Sizani et al., 2025)

## 7 Conclusion and future perspectives

The subfamily II of LRR-RLKs comprises versatile and evolutionarily conserved coreceptors such as BAK1, CIKs, and other SERKs that operate as nodes integrating developmental and immune signaling in plants. They are essential for receptor signaling and maintain the cognate receptor-specific molecular responses. In contrast, NIK1/2 may function as signaling hubs as they transduce different biotic and abiotic signals into a unique response, independently of the specific stimulus-sensing receptor. Whether NIK1/2 acts as a true coreceptor in a specific signaling pathway mediated by a cognate receptor remains to be determined. This review describes the emerging role of these coreceptors and signaling hubs as regulators of antiviral defenses at the interface with their roles in growth and growth responses. Their multifaceted functions are particularly evident in meristematic tissues, where growth–immunity trade-offs are tightly balanced, and viruses may hijack host signaling hubs to facilitate infection. LRRII-RLK members appear to play a role in maintaining cellular homeostasis both in the presence and absence of various stimuli, by either regulating each other or modulating mechanisms underlying growth and immunity.

SERKs have been intensively characterized, and a variety of cognate receptors have been identified. Additionally, intermediates of the receptor-mediated signaling pathway and the corresponding assembly of the receptor-specific response have been deciphered. In contrast, little is known about LRR2c members, and although some progress has been made in elucidating the NIK1/2-mediated signaling pathway, several questions remain unanswered. For instance, the viral PAMP recognition receptor has not been sorted out, and specific stress-sensing receptors have yet to be identified. Furthermore, we still do not know the kinases that specifically phosphorylate RPL10 and LIMYB. These gaps in the NIK1-RPL10-LIMYB signaling circuit need to be filled in.

NIK receptors represent a promising biotechnological target due to their central role in coordinating plant growth and defense processes. Strategies such as overexpressing constitutively active forms of NIK1 have already shown antiviral resistance in tomato, without severely compromising growth (Brustolini et al., 2015). Therefore, the potential generation of NIK1 variants resistant to NSP inhibition may offer a promising approach for developing begomovirus-resistant crops, especially in economically relevant crops. However, it is essential to carefully examine the potential adverse effects of NIK1-mediated suppression of translation and photosynthesis on each specific crop. For instance, in crops with high metabolic demands, a reduction in photosynthesis could lead to yield loss. Therefore, a crop-specific approach is required when considering NIK1 variant overexpression for antiviral resistance.

The existence of functionally redundant receptors may mitigate the adverse growth effects associated with constitutive pathway activation or hinder viral evasion mechanisms. Consequently, specific modulation of the antiviral function of each receptor represents a powerful tool for engineering more resilient plants. Recent studies have revealed diverse mechanisms by which plant viruses interfere with LRR-RLKII-mediated pathways, including targeting receptor complexes (e.g., BAM1-CIKs), modulating the expression of coreceptors (e.g., SERK4 in rice via P3), and neutralizing antiviral kinases (e.g., inhibition of NIK1–3 by NSP). These findings suggest that LRR-RLKII members are not passive agents but dynamic regulators. SERK1, BAK1, and NIK1/2 in Arabidopsis and NIK3 in cassava promote viral resistance, while SERK4 in rice is subverted to favor viral infection. In cassava, NIK3 expression is associated with CMD tolerance, while in rice, SERK4 has emerged as a susceptibility hub manipulated by RGSV. Remarkably, independent of their role as antiviral or proviral factors, all LRRII-RLKs are so far affected by a particular viral protein to favor viral infection.

Identifying the receptor(s) that perceive virus molecular patterns remains a central goal for understanding plant antiviral immunity. Additionally, examining ligand specificity and downstream signaling modules connected to the members of LRR-RLKII clades, such as the LRRII-C and CIK subgroups, is crucial for elucidating their roles as immune regulators. Structural studies may elucidate the basis of functional divergence between SERK paralogs in developmental versus immunological contexts. Understanding the coevolutionary dynamics between viruses and coreceptors, particularly the mechanisms by which viral proteins manipulate receptor function, is a fascinating area of research. Their functional plasticity and evolutionary conservation make them promising targets for biotechnological innovation in plant protection.

## Glossary

5′UTR: 5′ Untranslated Region
ACLSV: apple chlorotic leaf spot virus
ACR4: ARABIDOPSIS CRINKLY4
BAK1: BRI1-ASSOCIATED RECEPTOR KINASE
BAM: BARELY ANY MERISTEM
BES1: BRI-1-EMS-SUPRESSOR 1
BIK1: BOTRYTIS INDUCED KINASE 1
BIR: BAK1-INTERACTING RECEPTOR-LIKE KINASES
BRI: BRASSINOSTEROID INSENSITIVE
BKK1: BAK1-LIKE KINASE 1
BR: Brassinosteroid
CabLCV: cabbage leaf curl virus
CaMV: cauliflower mosaic virus
CERK1: CHITIN ELICITOR RECEPTOR KINASE 1
CIK: CLAVATA3 INSENSITIVE RECEPTOR KINASE
CK: Cytokinin
CLE: Embryo Surrounding Region
CLV: CLAVATA
CMD: cassava mosaic disease
CML41: CALMODULIN-LIKE 41
CMV: cucumber mosaic virus
CNGC17: CYCLIC NUCLEOTIDE-GATED ION CHANNEL 17
COI1: CORONANTINE INSENSITIVE 1
CoRK: Coreceptors
CP: Coat Protein
CRN: CORYNE
DAMP: Damage-Associated Molecular Pattern
DNA: Deoxyribonucleic Acid
dsvDNA: Double-Stranded Viral DNA
EDS1: ENHANCED DISEASE SUSCEPTIBILITY 1
EF-Tu: Elongation Factor Thermo unstable
EFR: EF-Tu RECEPTOR
EMS1: EXCESS MICROSPOROCYTES 1
ER: ERECTA RECEPTOR
ERL1: ERECTA-LIKE 1
ETI: Effector-Triggered Immunity
ExPRR: Extracellular Pattern Recognition Receptor
FER: FERONIA RECEPTOR
Flg22: Flagellina 22
FLS2: FLAGELLIN-SENSITIVE 2
FRK1: FLG22-INDUCED RECEPTOR-LIKE KINASE 1
HAMP: Herbivore-Associated Molecular Patterns
HSL2: HAESA-LIKE 2
IDA: INFLORESCENCE DEFICIENT IN ABSCISSION
InPRR: Intracellular Pattern Recognition Receptor
INR: INCEPTIN RECEPTOR
IOS1: IMPAIRED OOMYCETE SUSCEPTIBILITY 1
JA: Jasmonic Acid
LIMYB: L10-INTERACTING MYB DOMAIN
LRR: Leucine-Rich Repeat
LRR-RLK: Leucine-Rich Repeat-Receptor-Like Kinase
LYK5: LYSM DOMAIN RECEPTOR-LIKE KINASE 5
MAPK: Mitogen-Activated Protein Kinase
MAX2: MORE AXILLARY GROWTH 2
MP: Movement Protein
MYMV: mungbean yellow mosaic virus
NIK: NUCLEAR SHUTTLE PROTEIN-INTERACTING KINASE
NLR: Nucleotide-Binding Leucine-Rich Repeat Receptor
NSP: Nuclear Shuttle Protein
NRPD1: N-terminal regulatory protein of DEAD-box RNA helicase 1
ORMV: rapeseed mosaic virus
PAD4: PHYTOALEXIN DEFICIENT 4
PAMP: Pathogen-Associated Molecular Pattern
PBL1: PBS1-LIKE KINASE 1
PBS1: PROTEIN KINASE SUPERFAMILY PROTEIN
PEPR: PEP1 RECEPTOR
PD: Plasmodesmata
PDLP: PLASMODESMATA-LOCATED PROTEIN
PIK3IP1 (P3IP1): PHOSPHOINOSITIDE-3-KINASE-INTERACTING PROTEIN 1
PLT: PLETHORA
PM: Plasma Membrane
Poly(I:C): Polyinosinic: Polycytidylic Acid
PPV: plum pox virus
PR: Pathogenesis-Related Protein 1
PRR: Pattern Recognition Receptor
PSKR1: PHYTOSULFOKINE RECEPTOR
PTGS: Post-Transcriptional Gene Silencing
PTI: PAMP-Triggered Immunity
RAM: Root Apical Meristem
RD motifs: Arginine and Aspartate motif
RdDM: RNA-Directed DNA Methylation
RGSV: rice grassy stunt virus
RGI: RGF1 INSENSITIVE
RGF1: ROOT MERISTEM GROWTH FACTOR 1
RGFR: RGF RECEPTOR
RISC: RNA-Induced Silencing Complex
RK-CoR: Receptor Kinase- Co-receptor complex
RLK: Receptor-Like Kinase
RLCK: Receptor-Like Cytoplasmic Kinase
RLP: Receptor-Like Protein
RNA: Ribonucleic Acid
RNAi: RNA interference
ROS: Reactive Oxygen Species
RP: Ribosomal Protein
RPK2: RECEPTOR-LIKE PROTEIN KINASE 2
RPL10: RIBOSOMAL PROTEIN L10
SA: Salicylic Acid
SACMV: South African cassava mosaic virus
SAM: Shoot Apical Meristem
SERK: SOMATIC EMBRYOGENESIS RECEPTOR-LIKE KINASE
SCMV: sugarcane mosaic virus
SiRNA: Small Interfering RNA
SOBIR1: SUPPRESSOR OF BIR1-1
ssvDNA: Single-Stranded Viral DNA
TAIR: Arabidopsis Information Resource
TCP: TEOSINTE BRANCHED1
TCV: turnip shrivel virus
TGS: Transcriptional Gene Silencing
TMV: tobacco mosaic virus
TIR1: TRANSPORT INHIBITOR RESPONSE 1
TPD1: TAPETUM DETERMINANT 1
TRV: tobacco rattle virus
TSWV: tomato spotted wilt virus
TYLCV: tomato yellow leaf curl virus
vTALE: Viral Transcription Activator-Like Effector
WRKY29: WRKY DNA-BINDING PROTEIN 29
WUS: WUSCHEL
