# Supplementary material for: LRR-RLK subfamily II of coreceptors: emerging, non-canonical and canonical roles in plant antiviral immunity and development
Source: Front Plant Sci. 2025 Nov 17;16:1694090. doi: 10.3389/fpls.2025.1694090 (PMC12666859; doi:10.3389/fpls.2025.1694090)
Supplement: Supplementary file 1 [file DataSheet1.pdf]

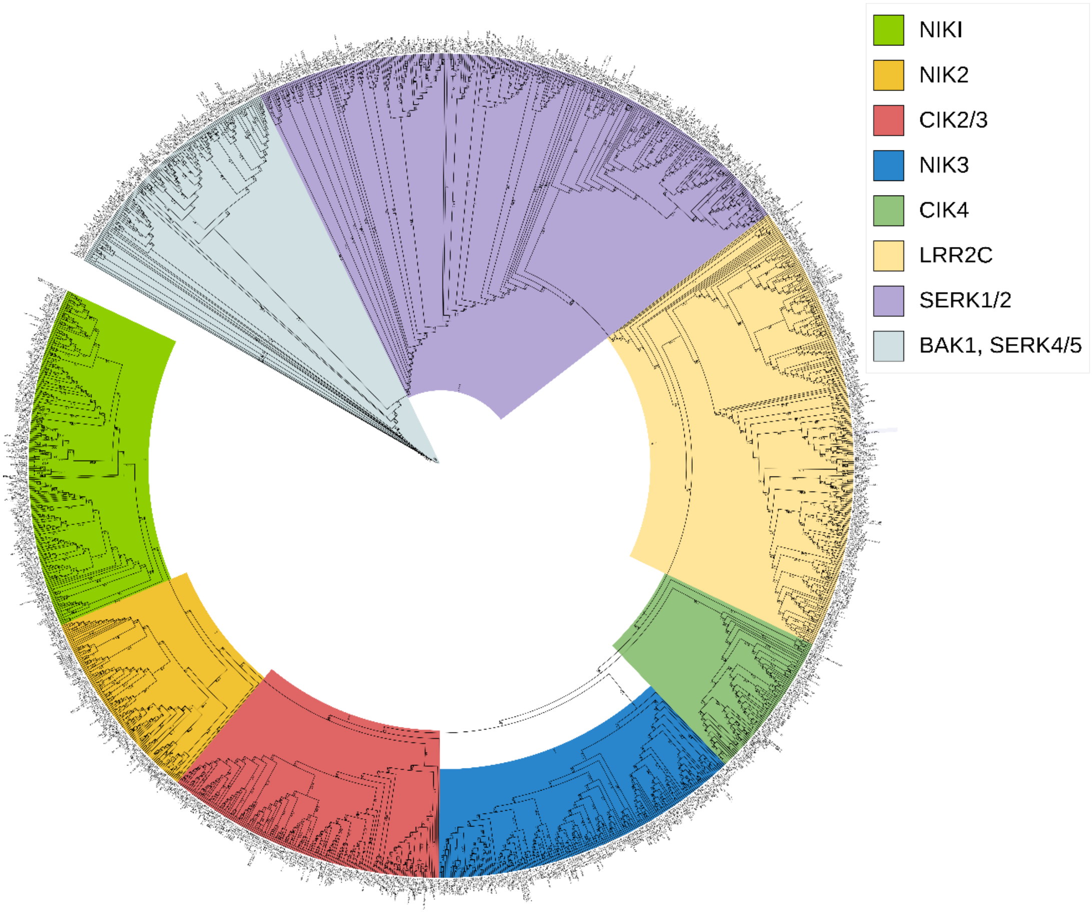

Supplementary Figure 1 – Phylogenetic analysis of LRR-RLK subfamily II orthologs across plant species. Protein sequences of the 14 *Arabidopsis thaliana* members of the LRR-RLK subfamily II were used as queries to identify orthologous genes in the PLAZA 5.1 database, retrieving 1,906 orthologs distributed across 94 plant species. Then, sequence trimming was performed with the trimAl v1.4.rev15 tool to reduce divergent regions with many gaps, with the -gappyout parameter. Subsequently, two statistical parameters were used, resoverlap 0.85 and -seqoverlap 80, which remove poorly conserved regions (< 85%) and sequences (< 80%), ensuring the presence of 10 more conserved sequences. This procedure resulted in 1,386 genes. Phylogenetic inference was performed with IQ-TREE2 v2.3.5 using the Maximum Likelihood (ML) statistical model, assuming the best substitution model Q.plant+R10 performed by the ModelFinder function. The phylogenetic method evaluated 1000 ultrafast bootstrap pseudoreplicates (-bb 1000) and 1000 replications of the SH-aLRT test (-alrt 1000). A file in Newick format (.nwk) was generated and visualized by the online tool Interactive Tree of Life (iTOL). See also https://itol.embl.de/export/20023519899154591760355171 for a better resolution.
