# Supplementary material for: LRR-RLK subfamily II of coreceptors: emerging, non-canonical and canonical roles in plant antiviral immunity and development
Source: Front Plant Sci. 2025 Nov 17;16:1694090. doi: 10.3389/fpls.2025.1694090 (PMC12666859; doi:10.3389/fpls.2025.1694090)
Supplement: Supplementary file 2 [file DataSheet2.pdf]

Supplementary Table 1 – *Arabidopsis thaliana* LRR-RLK *subfamily II.*

GENE ID	Other names	Description
AT1G34210	AtSERK2, SERK2	The plasma membrane LRR receptor-like serine/threonine kinase is expressed during embryogenesis in locules until stage 6 anthers, with higher expression in the tapetal cell layer. SERK1 and SERK2 receptor kinases function redundantly as an important control point for sporophytic development, controlling male gametophyte production. The mRNA is cell-to-cell mobile.
AT1G60800	AtNIK3, CIK1, NIK3	Encodes one of a group of LRR-RLKs, designated as CLAVATA3 INSENSITIVE RECEPTOR KINASES (CIKs), that act as coreceptors and have essential roles in regulating CLV3-mediated stem cell homeostasis.
AT1G71830	AtSERK1, SERK1	The plasma membrane LRR receptor-like serine/threonine kinase is expressed during embryogenesis in locules until stage 6 anthers, with higher expression in the tapetal cell layer. SERK1 and SERK2 receptor kinases function redundantly as a crucial control point for sporophytic development, regulating male gametophyte production. SERK1 interacts with and transphosphorylates EMS1
AT2G13790	AtSERK4, BAK7, BKK1, SERK4	Receptor-like protein kinase that plays a role in various processes, including the plant-type hypersensitive response, defense response, stomatal movement, and brassinosteroid signaling.
AT2G13800	AtSERK5, BAK8, SERK5	Pseudogen
AT2G23950	CIK2, CLERK	Encodes an LLR receptor kinase that is expressed in protophloem and is required for CLE peptide sensing in roots. One of a group of LRR-RLKs, designated as CLAVATA3 INSENSITIVE RECEPTOR KINASES (CIKs), that acts as a co-regulator and has essential roles in regulating CLV3-mediated stem cell homeostasis.
AT3G25560	AtNIK2, NIK2	NULL (ThaleMine) - Involved in defense response to geminivirus infection (By similarity); Phosphorylates RPL10A in vitro (UniProt)
AT4G30520	CIK3, SARK	Encodes SARK (SENESCENCE-ASSOCIATED RECEPTOR-LIKE KINASE). Regulates leaf senescence through synergistic actions of auxin and ethylene. It is one of a group of LRR-RLKs, designated as CLAVATA3 INSENSITIVE RECEPTOR KINASES (CIKs), that act as coreceptors and have essential roles in regulating CLV3-mediated stem cell homeostasis.
AT4G33430	AtBAK1, AtSERK3, BAK1, ELG, RKS10, SERK3	Leu-rich receptor Serine/threonine protein kinase. Component of BR signaling that interacts with BRI1 in vitro and in vivo to form a heterodimer. Brassinolide-dependent association of BRI1 and BAK1 in vivo. Phosphorylation of both BRI1 and BAK1 on Thr residues was BR-dependent. Although BAK1 and BRI1 alone are localized in the plasma membrane, when BAK1 and BRI1 are coexpressed, the heterodimer BAK1/BRI1 they form is localized in the endosome. Contributes to postinvasive immunity against Alternaria brassicola.
AT5G10290	-	-
AT5G16000	AtNIK1, NIK1	NSP-interacting kinase (NIK1), receptor-like kinase, involved in defense response against geminivirus. It acts as a virulence target of the begomovirus nuclear shuttle protein (NSP).

AT5G45780	CIK4	Encodes one of a group of LRR-RLKs, designated as CLAVATA3 INSENSITIVE RECEPTOR KINASES (CIKs), that act as coreceptors and have essential roles in regulating CLV3-mediated stem cell homeostasis.
AT5G63710	APEX	-
AT5G65240	-	-
